# Use of a short educational video to improve the accuracy of colorectal polyp morphology assessment: A multicenter randomized controlled study

**DOI:** 10.1002/deo2.70066

**Published:** 2025-02-03

**Authors:** Takahiro Utsumi, Takahiro Horimatsu, Yoshitaka Nishikawa, Akira Teramoto, Daizen Hirata, Mineo Iwatate, Shinwa Tanaka, Nobuaki Ikezawa, Masaya Esaki, Shozo Osera, Chikara Ebisutani, Hiroaki Saito, Nobukazu Agatsuma, Yukiko Hiramatasu, Yuki Nakanishi, Yasushi Sano, Hiroshi Seno

**Affiliations:** ^1^ Department of Gastroenterology and Hepatology Kyoto University Graduate School of Medicine Kyoto Japan; ^2^ Institute for Advancement of Clinical and Translational Science (iACT) Kyoto University Hospital Kyoto Japan; ^3^ Department of Therapeutic Oncology Kyoto University Graduate School of Medicine Kyoto Japan; ^4^ Department of Health Informatics Kyoto University School of Public Health Kyoto Japan; ^5^ Department of Gastroenterology and Hepatology Royal Brisbane and Women's Hospital Brisbane Australia; ^6^ Third Department of Internal Medicine Toyama University Hospital Toyama Japan; ^7^ Gastrointestinal Center and Institute of Minimally Invasive Endoscopic Care (iMEC) Sano Hospital Hyogo Japan; ^8^ Department of Internal Medicine Division of Gastroenterology Graduate School of Medicine Kobe University Hyogo Japan; ^9^ Department of Gastroenterology Handa City Hospital Aichi Japan; ^10^ Department of Gastroenterology Saku Central Hospital Advanced Care Center Nagano Japan; ^11^ Hiyodori Clinic Hyogo Japan; ^12^ Department of Internal Medicine Soma Central Hospital Fukushima Japan

**Keywords:** colonic polyps, education, internet, morphology, randomized controlled trial

## Abstract

**Objectives:**

Although accurate assessment of polyp morphology helps endoscopists select the appropriate management for colorectal polyps, some studies have reported unsatisfactory accuracy in such assessment. This study aimed to clarify the usefulness of a short educational video available on the Internet for accurate polyp morphology assessment.

**Methods:**

This was a multicenter randomized controlled trial. Participants were randomly assigned to the pre‐ or post‐education groups after a pre‐test comprising images of 42 polyps, including 12 laterally spreading tumors. Participants who scored ≥ 80% on the pre‐test were excluded. Only the post‐education group completed the diagnostic test after watching an online educational video. The primary outcome was the difference in diagnostic accuracy between the pre‐test and diagnostic tests for each group.

**Results:**

Of the 296 endoscopists enrolled from 48 institutions, 34 missed the test deadline, and 29 who scored ≥ 80% in the pre‐test were excluded. The primary outcome analysis sets were 117 and 116 in the pre‐ and post‐education groups, respectively. The mean pre‐test accuracies in the pre‐education and post‐education groups were 60.6% and 60.7%, respectively. The difference in diagnostic accuracy between the pre‐test and diagnostic test was significantly higher in the post‐education than the pre‐education group (12.0 points, 95% confidence interval [CI] 9.9–14.1 and 2.3 points, 95% CI 0.9–3.6; *p *< 0.001).

**Conclusion:**

This multicenter randomized controlled trial demonstrated the usefulness of a short educational video for accurate polyp morphology assessment.

## INTRODUCTION

Colorectal polyp removal reduces the incidence and mortality rates of colorectal cancer.^1^, ^2^ Accurate identification of polyp characteristics helps endoscopists predict the malignant potential of each lesion. Especially polyp morphology is an essential factor in determining the best treatment strategy, such as endoscopic therapy or surgery.^3^, ^4^ While depressed lesions tend to be highly invasive, pedunculated lesions are less likely to show invasive growth compared with sessile lesions.^5^, ^6^, ^7^ Pedunculated lesions have a low rate of lymph node metastasis even when they are early invasive cancers.^8^ Additionally, flat polyp morphology is associated with missed polyps or incomplete endoscopic resections.^9^, ^10^, ^11^ Morphological assessment using the laterally spreading tumors (LSTs) subclassification is also useful for predicting the malignant potential and invasion degree.^7^, ^12^ Therefore, Western and Japanese guidelines recommend assessing colorectal polyps using morphological classifications.^3^, ^4^, ^13^

In Japanese and Western studies, the Paris classification has been widely used to evaluate polyp morphologies.^5^, ^14^ However, despite its clinical importance, the diagnostic accuracy or inter‐observer agreement using this classification has not been fully assessed.^15^, ^16^, ^17^, ^18^ Additionally, in Japanese clinical practice, endoscopists often use the morphological classification by the Japanese Society for Cancer of the Colon and Rectum.^13^ Our previous study using the Japan Endoscopy Database clarified inter‐institutional differences in colorectal polyp morphology diagnosis in Japan.^19^ Even in Japan, where endoscopists are more interested in polyp morphology than in Western countries,^5^ the polyp morphology diagnosis is not accurate, even considering the possibility that the classification used was not uniform. Therefore, efforts to achieve a more accurate polyp morphology assessment are necessary.

Most endoscopists, including experienced instructors, have limited opportunities to receive appropriate feedback on their morphological diagnoses due to the subjective nature of polyp morphology assessment. This can lead to inaccuracies and disagreements in diagnosing polyp morphology between individuals and institutions. Although experience at our institution suggests that education could improve the accurate assessment of polyp morphology, there are few reports on education regarding polyp morphology, and its usefulness remains controversial.^15^, ^16^

Because the ability to precisely assess polyp morphology is critical for all endoscopists, the training modules must be simple and easy. Recently, education on endoscopic diagnosis using e‐learning systems has become widespread.^20^, ^21^, ^22^, ^23^ We developed a short video for polyp morphology assessment that is available on the Internet. Through this multicenter randomized controlled trial, we aimed to clarify the usefulness of video‐based training in improving the accuracy of polyp morphology diagnosis.

## METHODS

### Study design and participants

The present study was a multicenter randomized controlled trial. Gastrointestinal endoscopists from Japan were recruited between October and December 2021. The inclusion criteria were: all endoscopists who provided consent to participate in our study. All participants answered questions on their background and endoscopic experience, including sex (male/female), endoscopic experience (months), total number of colonoscopies conducted, number of colonoscopies conducted in the last year, and the presence of accreditation by the Japan Gastroenterological Endoscopy Society at enrollment. The exclusion criteria were as follows: (1) withdrawal of consent, (2) delay in answering the test questions, and (3) having an unresolved network problem when answering the test questions and watching the educational video. All participants were categorized as beginners, intermediates, and experts.^23^ Beginners were defined as endoscopists with < 3 years of endoscopic procedure experience or <300 colonoscopic procedures performed. In this study, the definition of “expert” was based on Japan Gastroenterological Endoscopy Society accreditation. The remaining endoscopists were categorized as intermediates. The study was conducted online. Written informed consent was obtained from all participants. The study protocol was approved by the institutional review board of Kyoto University Hospital, Kyoto, Japan (Approval number: C1482). The study was registered in a clinical trial registry (UMIN 000041285).

### Interventions and assignment

First, the participants were asked to take the pre‐test online. Participants distributed according to their pre‐test scores as an adjusting factor were randomly assigned to the pre‐ or post‐education groups with a 1:1 allocation, using random block sizes of four, six, and eight, stratified by the categories of beginners, intermediates, and experts. Randomization was performed with R (version 4.1.1) using the blockrand package (version 1.5). The participants were aware of their assigned group; however, the researchers involved in data collection and statistical analysis were all masked to the assignment.

As previously reported, participants who scored ≥ 80% in the pre‐test were excluded from the primary outcome analysis because they did not need further education.^21^ However, because the test results were not disclosed, they were also equally assigned. The educational video was accessible online to all participants for one month, from February 17 to March 16, 2022. Multiple viewings of the educational video were permitted, as its short length made it convenient for repeated viewing. Participants reported the number of times they viewed the educational video in the diagnostic test. Participants in the pre‐education group answered the diagnostic test within 2 weeks before the distribution of the educational video, and those in the post‐education group answered the diagnostic test within 2 weeks after the period during which the educational videos were available. Finally, 3 months after watching the short educational video, the participants undertook the post‐test to assess the sustainability of the video's educational effects. The correct answers for the tests were disclosed after the completion of the study.

### Pre‐test, diagnostic test, and post‐test

Our study included a pre‐test, an intervention (an educational video clip), a diagnostic test, and a post‐test. Tests of 42 randomly arranged cases, including 30 cases answering questions on polyp morphology according to the Paris classification and 12 cases answering questions regarding only the subtypes of LSTs, were prepared. The participants answered the polyp morphology by choosing from 12 options, including pedunculated (Ip), sessile (Is), slightly elevated (IIa), flat (IIb), slightly depressed (IIc), Ip + IIc, Is + IIc, IIa + IIc, Is + IIa, IIa + Is, IIc + IIa, and IIc + Is, and LST subtypes including granular homogenous (LST‐G‐H), granular nodular mixed (LST‐G‐NM), non‐granular flat elevated (LST‐NG‐FE), and non‐granular pseudo‐depressed (LST‐NG‐PD).^5^, ^7^, ^14^ When answering the polyp morphology questions, participants were asked to answer according to the Paris endoscopic classification; therefore, semi‐pedunculated polyps (Isp) were excluded. If the polyp had two different morphologies, the type occupying the largest area was answered first. To prevent misdiagnosis due to Japanese Society for Cancer of the Colon and Rectum classification use, the tests were designed so that the same answer would be given regardless of which classification was used, except that the Isp option was not available.

**FIGURE 1:**
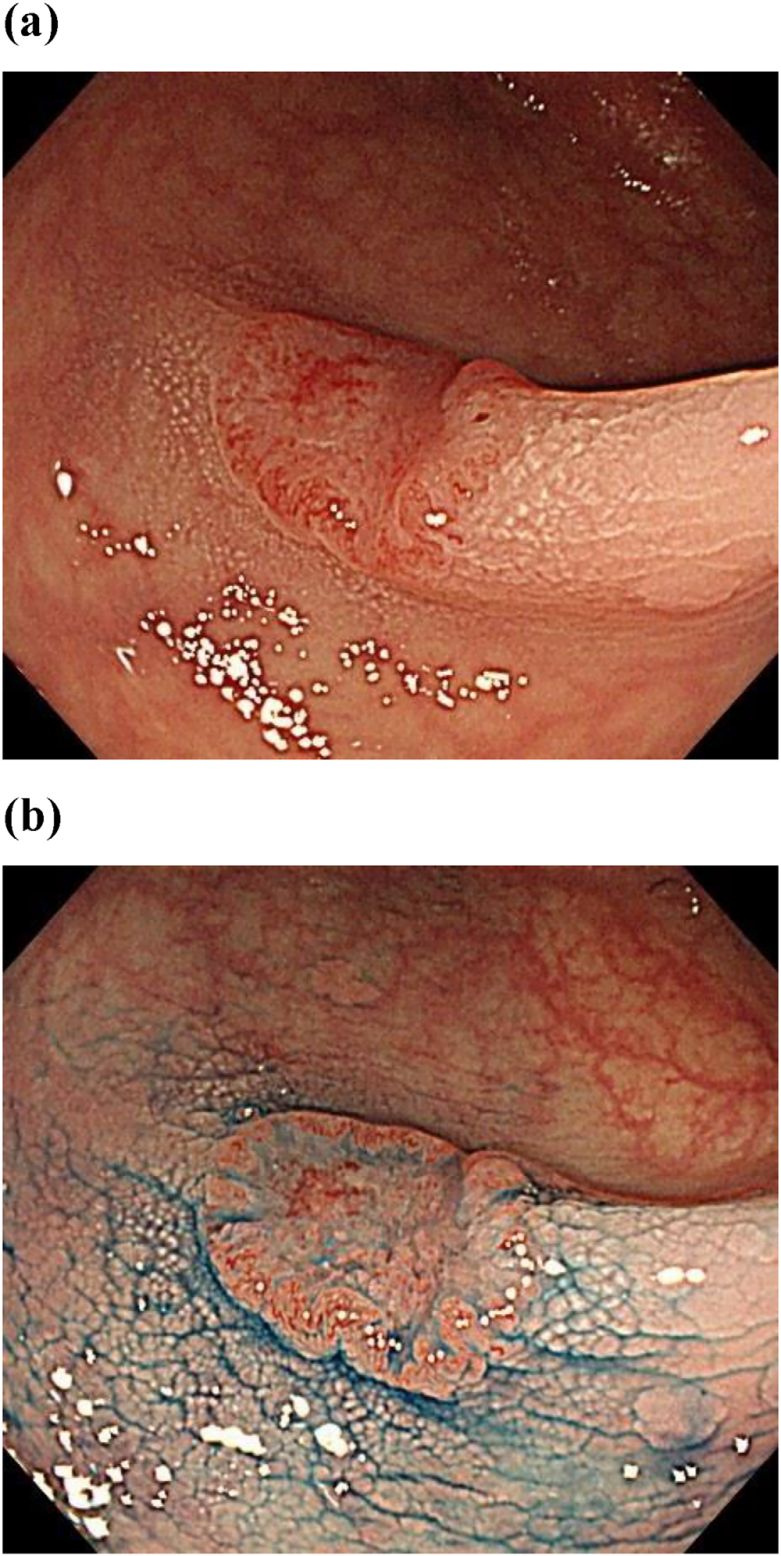
An example of the polyp images in the test. The test included images of 42 polyps, including 30 questions on polyp morphology according to the Paris classification and 12 questions on subtypes of laterally spreading tumors. Each pair of still images consisted of one distant image (Figure 1a) and one close image or image with indigo carmine (Figure 1b).

An example of the polyp case images in the tests is shown in Figure 1. A pair of still images consisting of one distant image and one close image or image with indigo carmine for each case was prepared at Sano Hospital and Kyoto University Hospital. Written informed consent was obtained from all patients regarding using their endoscopic images. Three expert endoscopists, members of the Japan NBI expert team,^24^ confirmed that the images used in the test were of sufficient quality to diagnose the morphology and LST subtypes. Table 1 shows the distribution of the polyps used in the test. The order of questions in the diagnostic test and post‐test was randomly rearranged from those in the pre‐test.

**TABLE 1:** Characteristics of the 42 lesions used in the tests.

Total polyps, *n*	42
Mean size^†^, mm (SD)	19.8 (18.2)
Category of size, *n* Diminutive/small/large	4/9/29
Shape, *n*	
Pedunculated (Ip)	3
Sessile (Is)	9
Flat elevated (IIa)	6
Flat depressed (IIc)	2
Complex type	10
LST‐G‐H	3
LST‐G‐NM	3
LST‐NG‐FE	3
LST‐NG‐PD	3
Histopathology, *n*	
LGD	26
HGIN	4
SM cancer	10
Serrated lesion	2

Abbreviations: HGIN, high‐grade dysplasia or adenocarcinoma confined to the mucosa; LST‐G‐H, laterally spreading tumor ‐ granular ‐ homogenous type; LST‐G‐NM, laterally spreading tumor, granular, nodular mixed type; LST‐NG‐FE, laterally spreading tumor‐non‐granular‐flat elevated type; LST‐NG‐PD, laterally spreading tumor‐non‐granular‐pseudo‐depressed type; LGD, low‐grade dysplasia; SD, standard deviation; SM, submucosal cancer.

^†^ The mean size of polyps, excluding the complex type and LSTs, was 8.7 mm (SD: 4.8).

### The educational video

**FIGURE 2:**
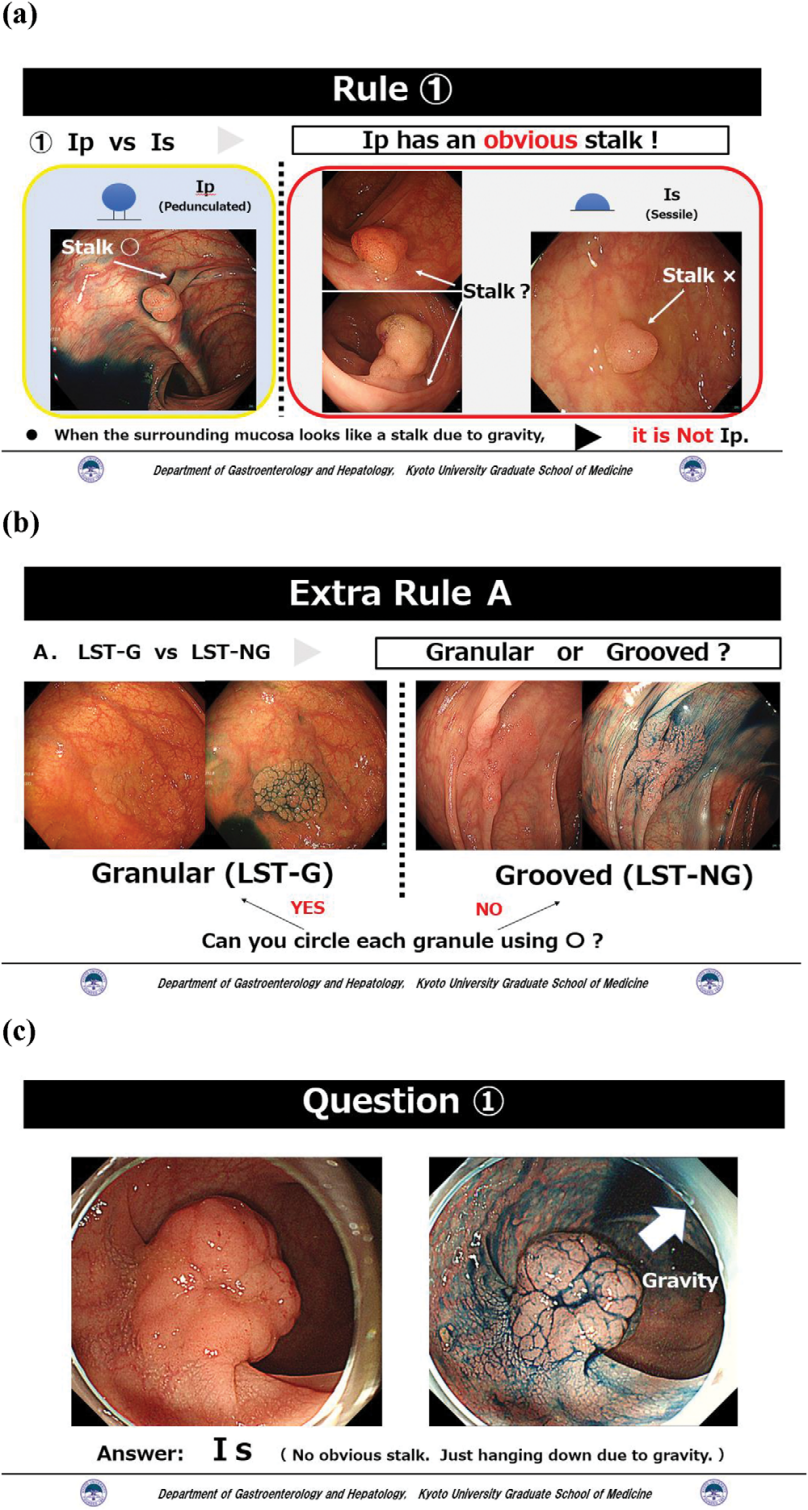
Examples of educational video content. Our educational video with an audio explanation was < 10 min long. First, the video introduced the Paris classification and the Japanese Society for Cancer of the Colon and Rectum guidelines’ classification, including the differences between the two classifications. Second, the course consisted of an explanation of the points where mistakes could easily be made for each type of morphology (a). Additionally, the video presented how to classify the subtypes of laterally spreading tumors with useful tips (b). Finally, the video included exercises with questions regarding polyp morphology and laterally spreading tumor subtypes (c).

The video lecture with audio explanations in Japanese was < 10 min long (Figure 2, Video S1, and Figure S1). The video introduced both classifications, including the Paris and the Japanese Society for Cancer of the Colon and Rectum guidelines’ classification. The course consisted of an explanation of the differences between the two classifications and the points where mistakes could easily be made in each type of morphology according to the Paris classification. In addition, the video allowed learning the LST subtype classification and exercise with questions about polyp morphology and LST subtypes.

### Outcomes

#### Primary outcome measure

The primary outcome measure was the difference in diagnostic accuracy between the pre‐test and diagnostic tests in the pre‐ and post‐education groups.

#### Secondary outcome measures

Secondary outcome measures were: 1) the changes of accuracy in each morphology and subtype identification between the pre‐test and diagnostic test in the post‐education group; 2) changes in accuracy rate between the diagnostic test and post‐test in the post‐education group for investigating whether the educational effect is maintained; 3) multivariate analysis of factors that contribute to the educational effectiveness of the short educational video in the post‐education group with high‐score (≥ 80%) participants.

### Statistical analysis

As no morphology assessment accuracy data were available in our study setting and there were no expected disadvantages to the participants, we invited as many participants as possible without setting a sample size. The accuracy of each test was compared by using paired t‐tests. The range of confidence intervals (CIs) was calculated based on the results of each participant. Continuous and categorical variables were compared using the chi‐squared or Mann–Whitney *U* tests. Univariate and multivariable logistic regression analyses of factors that contributed to an increase of ≥10% in accuracy were conducted based on the classification of endoscopists, sex, number of colonoscopies conducted in the last year, pre‐test score (%), and number of educational video views. Differences were considered statistically significant at *p* < 0.05. All statistical analyses were performed using IBM SPSS software (version 24.0; IBM Corp.).

## RESULTS

### Participants and characteristics

Among 296 enrolled endoscopists from 48 institutions, 33 missed the pre‐test deadline and 29 who scored ≥ 80% in the pre‐test were excluded. Of the 234 participants, 117 were allocated to the pre‐education group and 117 to the post‐education group. Participants with high scores (≥80%) were also randomized to each group to investigate factors that contribute to the effectiveness of the educational video.

**FIGURE 3:**
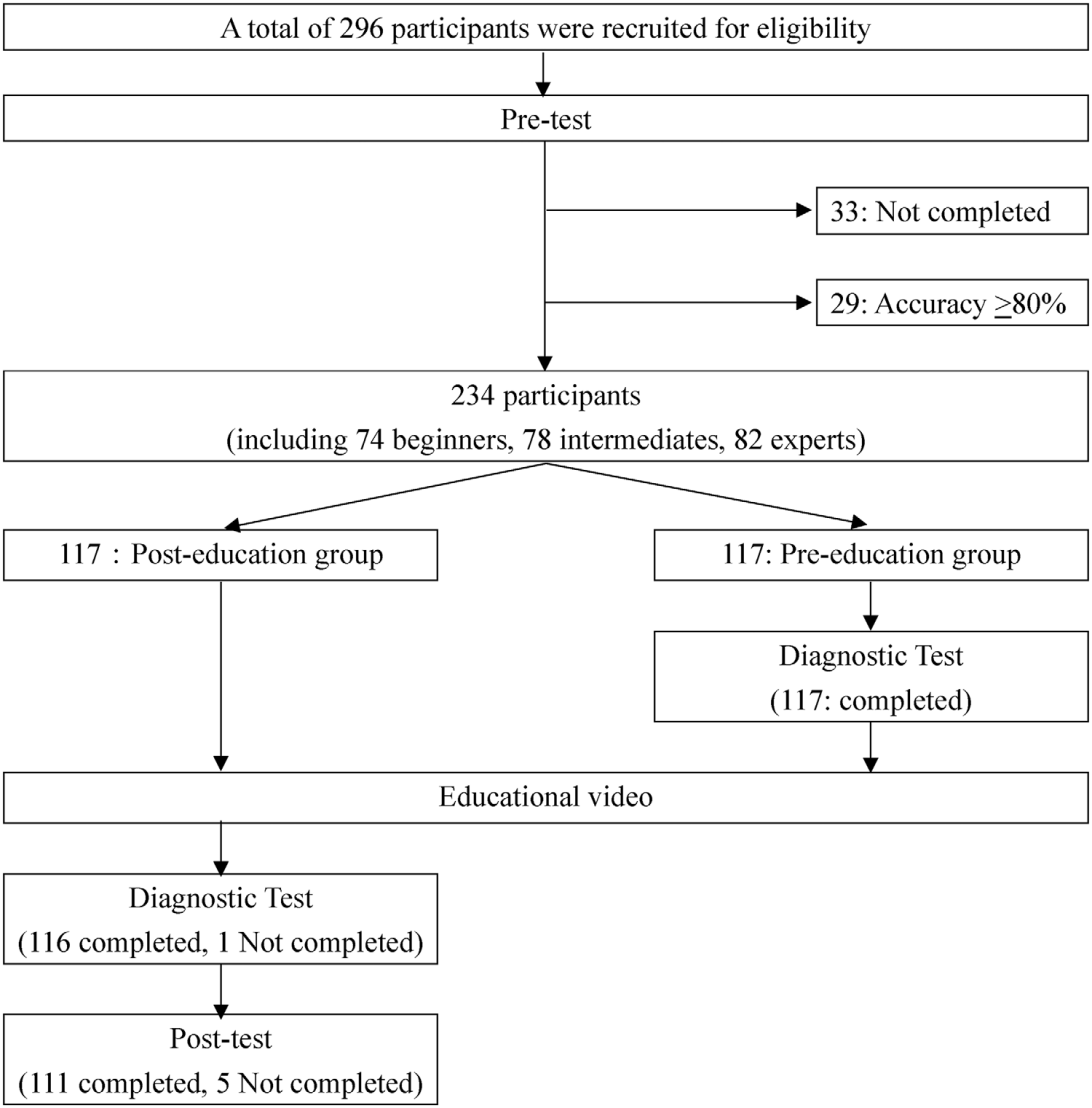
Flow chart of participant enrollment.

In the post‐education group, one participant did not answer the diagnostic test, and five did not answer the post‐test. In the pre‐education group, all the participants completed the diagnostic test. The participants had no trouble viewing the educational video on the Internet. There were no missing values in the test results and no unexpected disadvantages to the participants. The analysis set for the primary outcome included 117 pre‐education and 116 post‐education groups (Figure 3). Table 2 shows each group's characteristics.

**TABLE 2:** Characteristics of the 233 participants, including pre‐education and post‐education groups.

	Pre‐education group^†^ (n = 117)	Post‐education group^†^ (n = 116)	*p‐*value
Sex, *n*			
Male/female	94/23	90/26	0.63
Classification of endoscopists, *n*			
Beginners/intermediates/experts^‡^	37/39/41	36/39/41	1.00
Months of endoscopic experience, mean (SD), *n*	90.9 (76.8)	92.9 (84.6)	0.87
Total number of colonoscopies conducted, mean (SD), *n*	1935 (2430)	2009 (2880)	0.72
Number of colonoscopies conducted in the last year, mean (SD), *n*	198 (156)	209 (166)	0.62
Pre‐test score, mean (SD), %	60.6 (12.2)	60.7 (12.0)	1.00

^†^ Participants in the pre‐education group completed the diagnostic test before the educational video was distributed, while those in the post‐education group completed the test after watching the educational video.

^‡^ Beginner group included endoscopists with <3 years of experience in endoscopic procedures or < 300 colonoscopic procedures. The expert group was defined as endoscopists accredited by the Japan Gastroenterological Endoscopy Society. The remaining endoscopists were categorized into the intermediate group.

SD, standard deviation.

### Outcomes

#### Primary outcome measure

The mean accuracy in the pre‐education group was 60.6% (95% CI 58.3%–62.8%) and 62.9% (95% CI 60.7%–65.0%) for the pre‐test and diagnostic test, respectively. In contrast, the accuracies in the pre‐test and diagnostic test in the post‐education group were 60.7% (95% CI 58.4%–62.9%) and 72.7% (95% CI 70.6%–74.7%), respectively (Table 3). The differences in diagnostic accuracy between the pre‐test and diagnostic test were significantly higher in the post‐education group than in the pre‐education group (12.0 points, 95% CI 9.9–14.1; vs 2.3 points, 95% CI 0.9–3.6; *p* < 0.001; Table 4).

**TABLE 3:** Changes in accuracy between the pre‐test and post‐test in the 117 pre‐education and 116 post‐education participants.

	Pre‐test (95% CI)	Diagnostic test (95% CI)	Changes^‡^
Pre‐education group^†^ (*n* = 117)	60.6% (58.3–62.8%)	62.9% (60.7–65.0%)	+2.3 points (+0.9‐+3.6 points)
Post‐education group^†^ (*n* = 116)	60.7% (58.4–62.9%)	72.7% (70.6–74.7%)	+12.0 points (+9.9‐+14.1 points)

Abbreviation: CI, confidence interval.

^†^ Participants in the pre‐education group completed the diagnostic test before the educational video was distributed, while those in the post‐education group completed the test after watching the educational video.

^‡^ The changes in diagnostic accuracy between the pre‐test and diagnostic test were significantly higher in the post‐education group than in the pre‐education group (*p *< 0.001).

**TABLE 4:** Changes in accuracy between the pre‐test, diagnostic test, and post‐test in the 111 post‐education group participants.

	Pre‐test^‡^ (95% CI)	Diagnostic test (95% CI)	Post‐test^‡^ (95% CI)
Post‐education group^†^ (*n* = 111)	60.9% (58.7–63.2%)	72.9% (70.9–75.0%)	70.9% (68.9–72.9%)

Abbreviation: CI, confidence interval.

^†^ Of the 116 participants in the post‐education group, 111 endoscopists answered all the tests, including the pre‐test, diagnostic test, and post‐test.

^‡^ Accuracy was significantly higher in the post‐test than in the pre‐test (*p* < 0.001).

#### Secondary outcome measures

Table 5 and Table S1 show the changes in accuracy for each morphology and subtype between the pre‐test and diagnostic test in the post‐education group. Diagnostic accuracy increased for most morphologies and LST subtypes except for questions using the image of a pedunculated polyp (Ip). The sensitivity, specificity, and negative predictive value for differentiating between Ip and non‐Ip were 95.1%, 89.6%, and 99.4%, respectively, in the pre‐test, and 90.5%, 95.1%, and 98.9%, respectively, in the diagnostic test.

**TABLE 5:** Changes in accuracy between pre‐test and diagnostic test, according to polyp morphology, including laterally spreading tumors.

	Pre‐test accuracy	Diagnostic test accuracy	Changes
Pedunculated (Ip) *n* = 6	95.1%	90.5%	‐ 4.6 points
Sessile (Is) *n* = 9	62.4%	78.6%	+ 16.2 points
Flat elevated (IIa) *n* = 3	34.8%	64.2%	+ 29.4 points
Flat depressed (IIc) *n* = 2	78.9%	85.8%	+ 6.9 points
Complexed type *n* = 10	40.1%	51.8%	+ 11.7 points
LST *n* = 12	77.8%	83.1%	+5.3 points

Abbreviation: LST, laterally spreading tumor.

Changes in the accuracy rate between the diagnostic test and post‐test in the post‐education group were investigated to assess whether the educational effect was maintained. In the post‐education group, 111 participants who completed the post‐test were analyzed for this outcome. The mean scores were 60.9% (95% CI 58.7%–63.2%), 72.9% (95% CI 70.9%–75.0%), and 70.9% (95% CI 68.9%–72.9%) in the pre‐test, diagnostic test, and post‐test, respectively. The accuracy in the post‐test was significantly higher than in the pre‐test (9.9 points, 95% CI 7.8–12.1; *p* < 0.001; Table 4).

To analyze the factors that contributed to an increase in accuracy, 130 participants with 16 high‐score (≥ 80%) participants in the post‐education group were analyzed, classified into the group of ≥10 points (improvement group) or < 10 points (non‐improvement group) in the accuracy. Table 6 summarizes each group's characteristics. Univariate analysis showed differences in the two groups’ number of views of educational videos and pre‐test scores. In the multivariate logistic regression analysis, only pre‐test scores (%) were significantly associated with an increase in accuracy (odds ratio 0.90, 95% CI 0.87–0.94, *p* < 0.001; Table 7). The result was similar when excluding participants with pre‐test scores above 80% (Table S2). Sensitivity analysis showed that 22 participants with a pre‐test score ≤ 50% increased from 42.0% to 63.0%, 32 with a score > 50% and ≤ 60% increased from 55.4% to 71.5%, respectively, in accuracy.

**TABLE 6:** Characteristics of the participants according to the improvement of accuracy after the educational video lecture.

	Endoscopists with the improvement (≥10 points) (*n* = 62)	Endoscopists with the improvement (<10 points) (*n* = 68)	*p‐*value
Sex, *n*			
Male/female	47/15	54/14	0.62
Classification of endoscopists, *n*			
Beginners/intermediates/experts^†^	19/22/21	18/22/28	0.69
Number of colonoscopies conducted in the last year, mean (SD), *n*	215 (186)	227 (158)	0.35
Number of views of the educational video, *n*	215 (186)	227 (158)	0.35
Pre‐test score, mean (SD), %	56.5 (12.5)	69.2 (11.4)	< 0.001
Diagnostic test score, mean (SD), %	77.1 (10.3)	71.2 (11.7)	0.004

^†^ The beginner group included endoscopists with < 3 years of experience in endoscopic procedures or < 300 colonoscopic procedures. The expert group was defined as endoscopists accredited by the Japan Gastroenterological Endoscopy Society. The remaining endoscopists were categorized into the intermediate group.

SD, standard deviation.

**TABLE 7:** Multivariate analysis of factors that contributed to the improvement (≥10%) in accuracy after the educational video lecture.

Variable	β	OR (95% CI)	*p*‐value
Classification of endoscopists			
Non‐beginners	0.69	2.00 (0.76–5.29)	0.16
Beginners	Reference	Reference	
Sex			
Male	Reference	Reference	1.00
Female	−0.003	1.00 (0.36–2.79)	
Number of colonoscopies conducted in the last year (*n*)	0.002	1.00 (1.00–1.00)	0.14
Pre‐test score (%)	−0.10	0.90 (0.87–0.94)	<0.001
Number of views of the educational video (n)	0.51	1.67 (0.87–3.20)	0.12

Abbreviations: β, Regression coefficient; CI, confidence interval; OR, odds ratio.

## DISCUSSION

We developed a simple educational video (<10 min) that is easily accessible on the Internet to improve the accuracy of colorectal polyp morphology assessment. The present multicenter randomized controlled trial, which included 234 endoscopists with various colonoscopic experience levels, demonstrated the usefulness of the educational video on polyp morphology assessment. Additionally, the improved accuracy of polyp morphology assessment remained high even 3 months after the education.

Our educational video is the first training module on polyp morphology to demonstrate its usefulness in a randomized controlled trial. Although the Paris classification is the most commonly used international classification system for determining polyp morphology, some studies have shown that the accuracy and inter‐observer and/or intra‐observer agreement to diagnose polyp morphology using this classification were unsatisfactory.^15^, ^16^, ^17^, ^18^ To improve diagnostic performance, classifications simpler than the Paris classification and training models for accurate morphology diagnosis have been proposed.^15^, ^16^, ^17^ A simplified classification may increase diagnostic concordance; however, its usefulness in clinical practice is unclear. Few studies have investigated whether education can improve the diagnostic performance of polyp morphology assessments.^15^, ^16^ Additionally, these studies were either small in size or conducted at a single institution. Research using the Japan Endoscopy Database has clarified the inter‐institutional differences, as well as inter‐observer differences in the diagnosis of colorectal polyp morphology.^19^ Our trial, targeting 234 endoscopists from 48 institutions, clarified the usefulness of training models for polyp morphology assessment, regardless of endoscopist backgrounds, including endoscopic experience and institution.

The multivariate logistic regression analysis showed that the only factor associated with a high improvement (≥10 points) in accuracy after the educational video was a lower score in the pre‐test, suggesting that the education was effective, particularly for endoscopists who had lower accuracy, regardless of endoscopic experience. The widespread use of educational videos, not only for beginners but also for experts, can help solve inaccuracies in diagnosing polyp morphology among institutions or individuals. However, the accuracy of Ip lesion identification decreased slightly from the pre‐test to the diagnostic test. Worsening of sensitivity and improving specificity for differentiation of Ip from non‐Ip suggest that the educational video helped endoscopists determine the diagnosis of Ip more carefully. Whether pedunculated early invasive colorectal cancers pathologically diagnosed as head invasion can be managed by endoscopic treatment is controversial.^8^, ^25^, ^26^ Thus, the influence of polyp morphology assessment on treatment strategy selection would be small.

Our educational video's strengths in improving the polyp morphology assessment include accessibility and easily shareable content. Educational videos available online have significant advantages over other teaching methods. The effect of an educational module on diagnostic performance depends largely on the content. Due to its simplicity, we could present the content as a supplement. The video can be viewed several times within a short period through the Internet. Additionally, despite its ease of viewing, the educational effect was well‐maintained even 3 months later.

This study had some limitations. First, it remains unclear how the videos will improve the decision‐making process for treatment. Its usefulness needs to be validated in a clinical setting. After viewing the video, it would be beneficial to assess whether the inter‐institutional discrepancy in morphological diagnoses, particularly for polyps with depressed areas or pedunculated polyps, the diagnosis of which could directly influence the treatment strategy, can be improved in settings similar to our previous study using the Japan Endoscopy Database.^19^ Second, still images of the polyps were used in the tests, which differs from clinical practice. However, if endoscopists can accurately diagnose polyp morphology using still images, that contain less information, polyp morphology diagnosis in clinical practice could be more accurate. Third, prior to the diagnosis of LST subtypes, it is essential to determine that the polyp measures ≥ 10 mm. Therefore, the educational effect on LST classification may be limited. Finally, whether our educational video can improve inter‐observer agreement with the Paris classification is uncertain. The unsatisfactory agreement reported by previous studies suggests the need for improvement in inter‐observer agreement and accuracy. However, a higher accuracy of polyp morphology assessment is expected to improve inter‐observer agreement.

In conclusion, our short educational video (<10 min) on polyp morphology significantly improved the accuracy of colorectal polyp morphology assessment. Furthermore, the educational effect lasted for at least 3 months after education. This simple and accessible module allows endoscopists to diagnose polyp morphology more accurately, leading to selecting the best strategy for colorectal polyp treatment.

## CONFLICT OF INTEREST STATEMENT

None.

## ETHICS STATEMENT

The study protocol was approved by the institutional review board of Kyoto University Hospital, Kyoto, Japan (Approval number: C1482).

## PATIENT CONSENT STATEMENT

All participants provided written informed consent in our study.

## CLINICAL TRIAL REGISTRATION

The study was registered in a clinical trial registry (UMIN 000041285).

## Supporting information



FIGURE S1 The slides used in the educational video.

TABLE S1. Changes in pre‐test and diagnostic test accuracy in the complexed type and the subtype of laterally spreading tumors.
TABLE S2. Multivariate analysis of factors that contributed to accuracy improvement (>10%) after the educational video lecture among participants, excluding those with pre‐test scores >80%.

Video S1. The educational video to improve the accuracy of colorectal polyp morphology assessment (Japanese version).
