# Supplementary material for: Use of a short educational video to improve the accuracy of colorectal polyp morphology assessment: A multicenter randomized controlled study
Source: DEN Open. 2025 Feb 3;5(1):e70066. doi: 10.1002/deo2.70066 (PMC11791016; doi:10.1002/deo2.70066)
Supplement: Supplementary file 1 — FIGURE S1 The slides used in the educational video. [file DEO2-5-e70066-s001.pdf]

Educational video
for
Colorectal polyp morphology

CONTENTS

1. Introduction – Classification of morphology -

2. Rules for polyp morphology assessment

3. Exercises

Classification of polyp morphology

✓ **There are two classifications of polyp morphology.**

1. **The classification by JSCCR[1)]**

2. **Paris classification （international classification）**

    ・ **This Classification was designed to facilitate easy diagnose.**

[1)]Japanese Society for Cancer of the Colon and Rectum

JSCCR classification

Protruded type

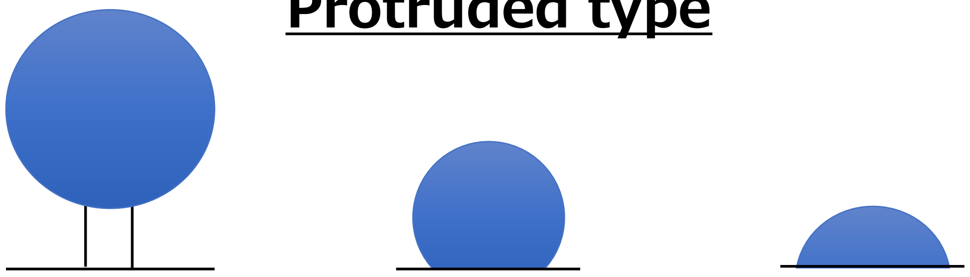

Ip (pedunculated)

Isp (semi-pedunculated)

Is (sessile)

Ip: Polyp with a stalk

Isp: Spherical polyp, a part of which is attached to the wall

Is: Hemispherical polyp, the base of which is attached to the wall

Superficial type

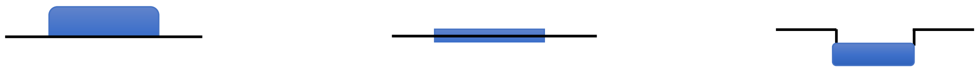

IIa (elevated)

IIb (flat)

IIc (depressed)

IIa: Flat and elevated lesion with a smooth surface

IIb: Flat lesion with almost the same height as the surrounding mucosa

IIc: Flat and depressed lesion lower in height than the surrounding mucosa

【III can be ignored in the classification of colonic polyps.】

JSCCR classification

Protruded type

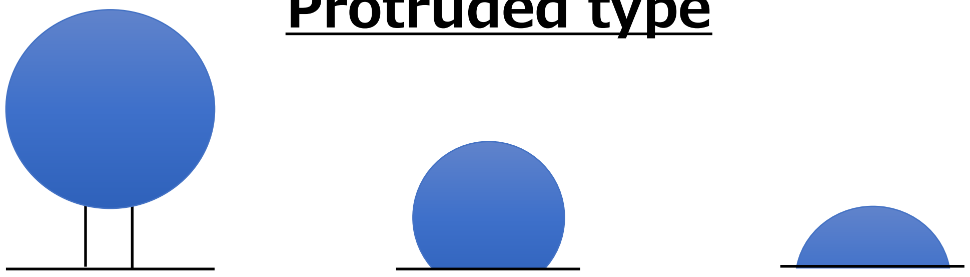

I[illegible]

(pedu[illegible]

Ip: Polyp with a stalk

Isp: Spherical polyp, a part of which is attached to the wall

Is: [illegible]

Diagnoses based on this criterion can be abstract or subjective.

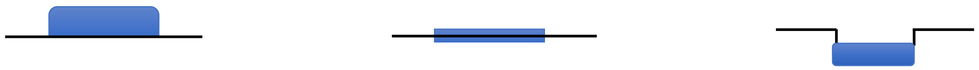

IIa (elevated)

IIb (flat)

IIc (depressed)

IIb: Flat lesion with almost the same height as the surrounding mucosa

IIc: Flat and depressed lesion lower in height than the surrounding mucosa

【III can be ignored in the classification of colonic polyps.】

Paris classification

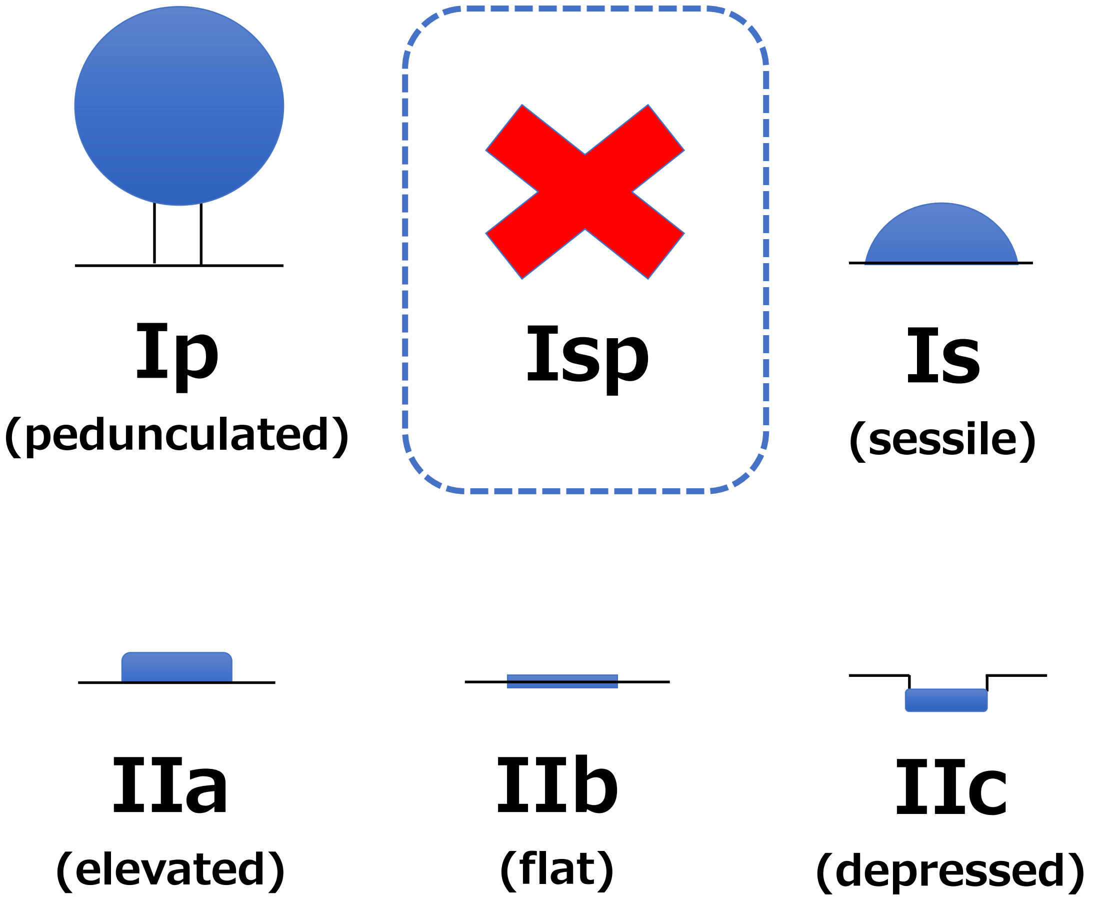

【III can be ignored in the classification of colonic polyps.】

When the height is more than 2.5mm,

With a stalk → **Ip** (pedunculated)

Without a stalk → **Is** (sessile)

Paris classification does not include Isp to avoid ambiguity.

IIa: Superficial elevated lesion less than 2.5mm in height

IIb: Superficial lesion, completely flat (very rare in colorectal lesion)

IIc: Superficial depressed lesion that is at least 1.2 mm lower than the surrounding mucosa

Paris classification

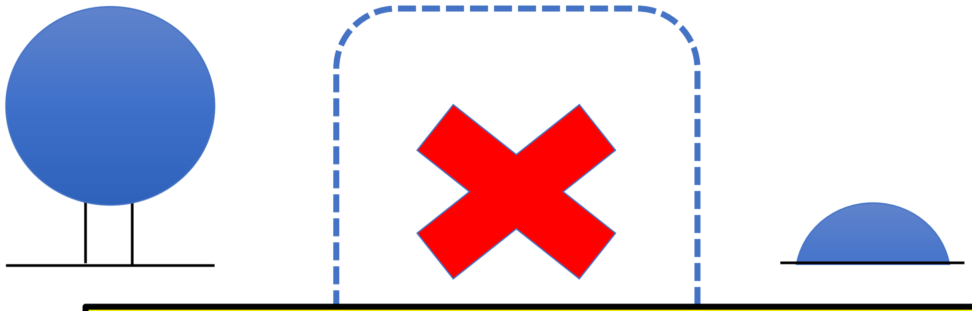

(pedun

When the height is more than 2.5mm,

With a stalk → **Ip** (pedunculated)

Without a stalk → **Is** (sessile)

guity.

Diagnoses based on this criterion can be more objective.

nm

in height

IIb: Superficial lesion, completely flat
(very rare in colorectal lesion)

IIc: Superficial depressed lesion that is at least 1.2 mm lower than the surrounding mucosa

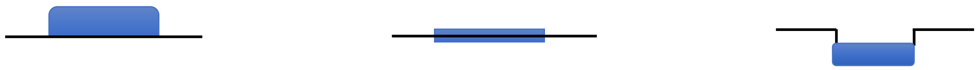

**IIa**	**IIb**	**IIc**
(elevated)	(flat)	(depressed)

【III can be ignored in the classification of colonic polyps.】

Morphological assessment in Japan

✓ **The proportion of each morphological type of polyps diagnosed in the top-10 facilities in the Japan Endoscopy Database (JED).**

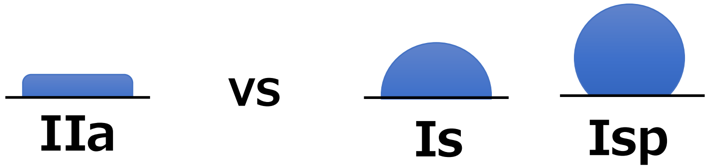

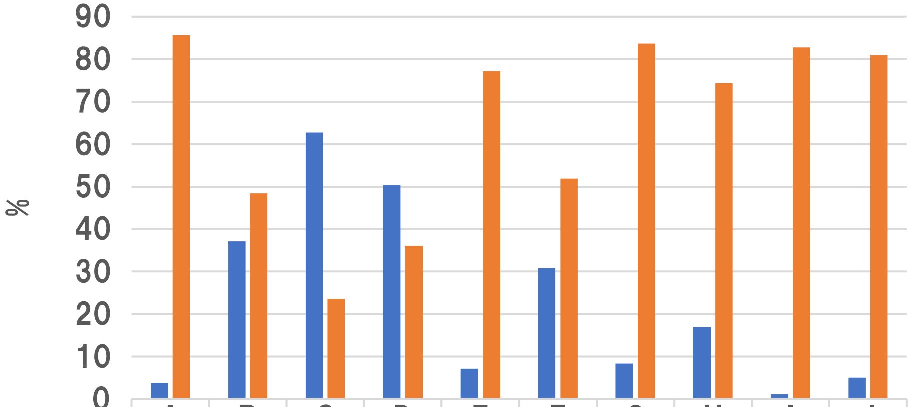

	A	B	C	D	E	F	G	H	I	J
IIa	3.8	37.1	62.7	50.4	7.1	30.8	8.4	17.0	1.2	5.1
Is/Isp	85.7	48.5	23.6	36.1	77.2	51.9	83.6	74.3	82.7	81.0

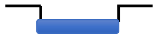

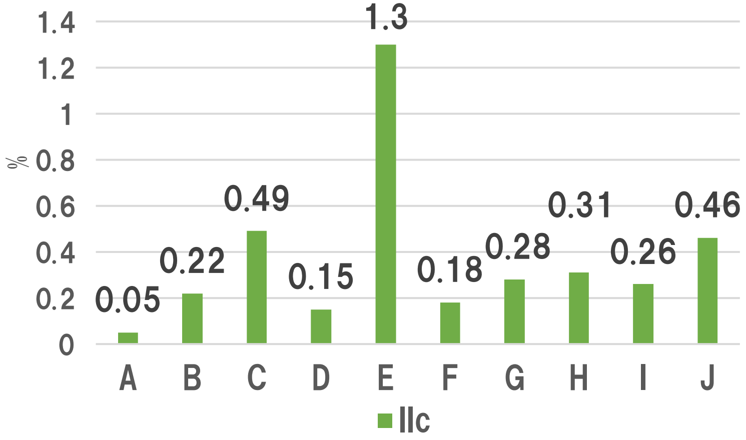

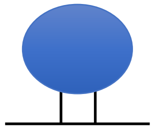

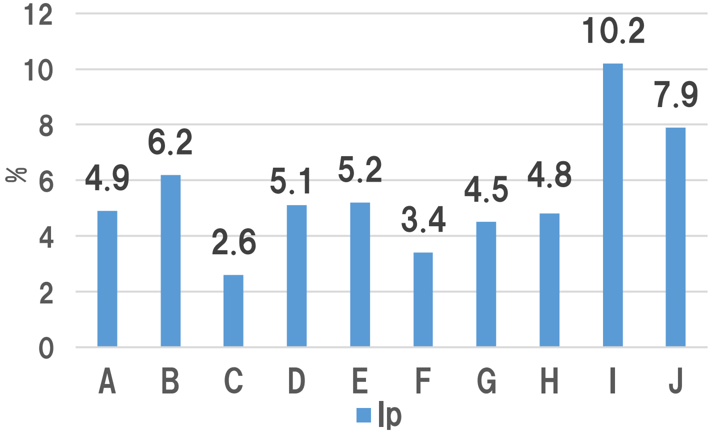

Utsumi T, et al. EJGH 2021.

The JED clarified inter-institutional differences in the diagnosis of polyp morphology.

Task

✓ **Efforts to achieve more accurate assessments of polyp morphology are necessary.**

Focusing also on increasing the agreement rate,
this video will be explained using the Paris Classification.

Rule ①

① Ip vs Is

Ip has an obvious stalk !

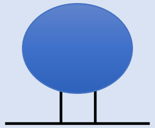

Ip
(Pedunculated)

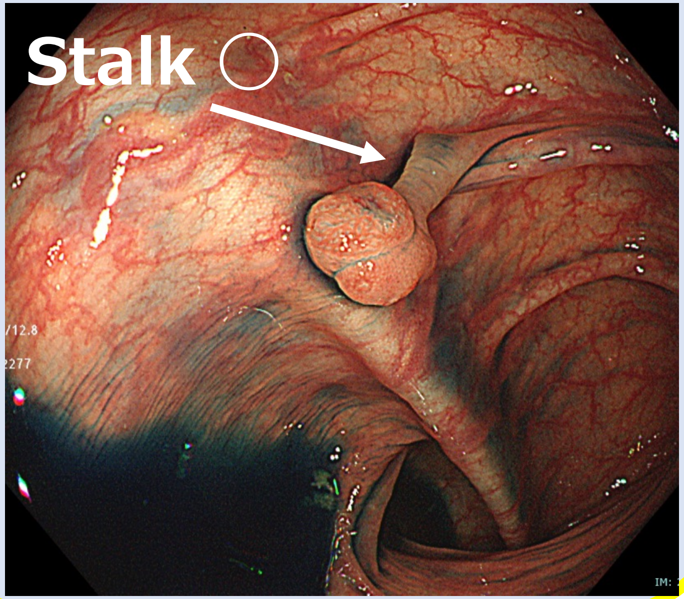

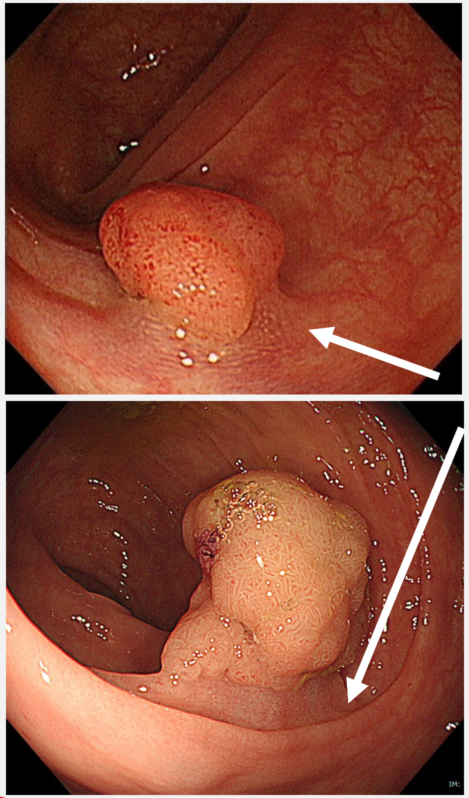

Stalk ?

Is
(Sessile)

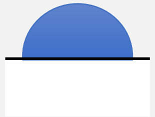

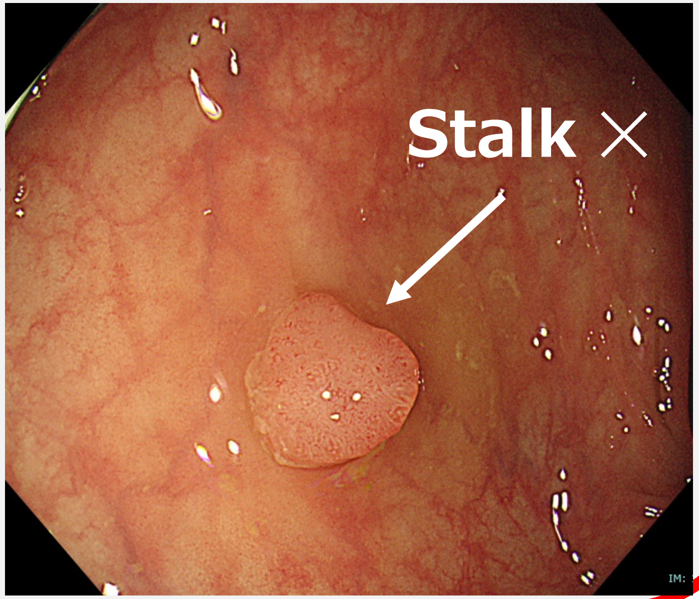

- When the surrounding mucosa looks like a stalk due to gravity, ▶ It is **Not** Ip.

Department of Gastroenterology and Hepatology, Kyoto University Graduate School of Medicine

Rule ①

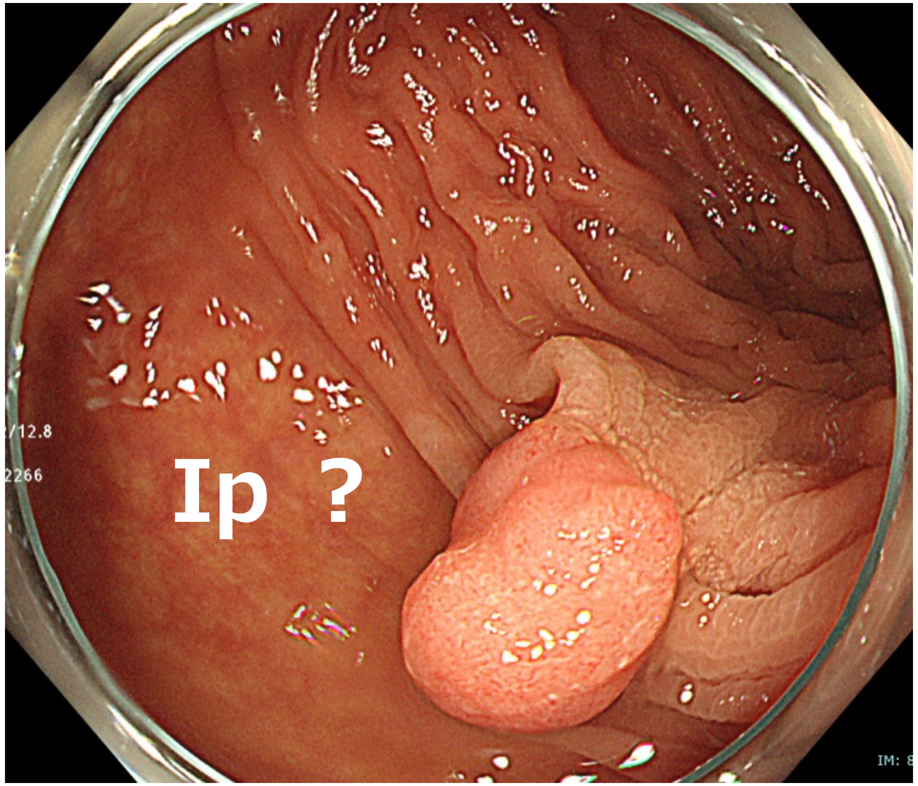

insufflation

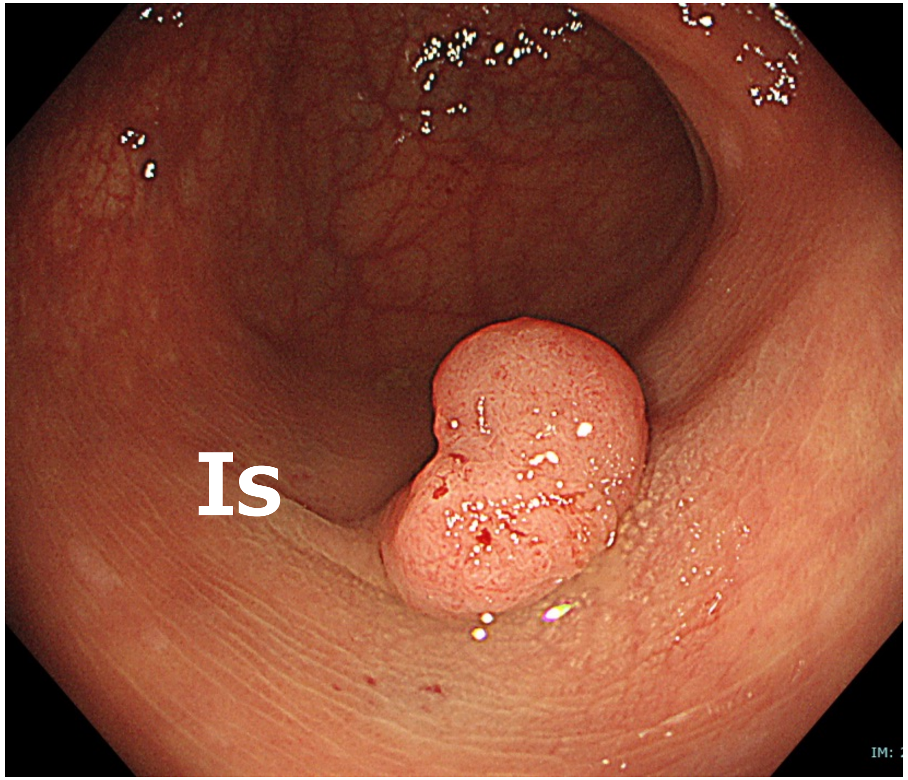

Do not rely solely on the images taken during deaeration.

Determine whether a stalk exists during insufflation. ▶ **If one is unsure of the presence of a stalk, it is not Ip.**

Rule ②

② Is vs IIa ▶ Refer to a device diameter of 2.5mm !!

Is
(Sessile)

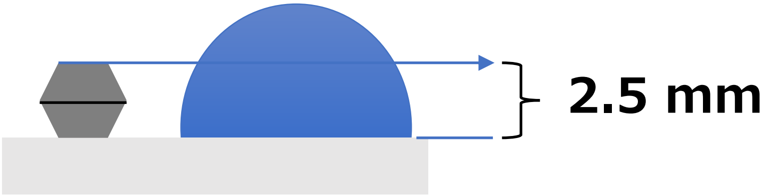

IIa
(Flat elevated)

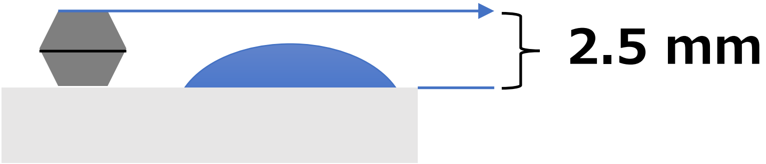

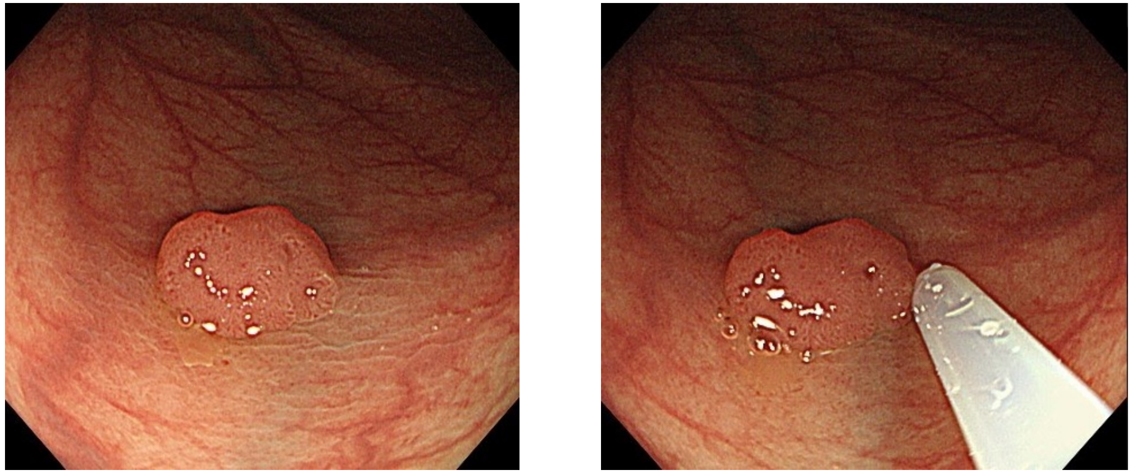

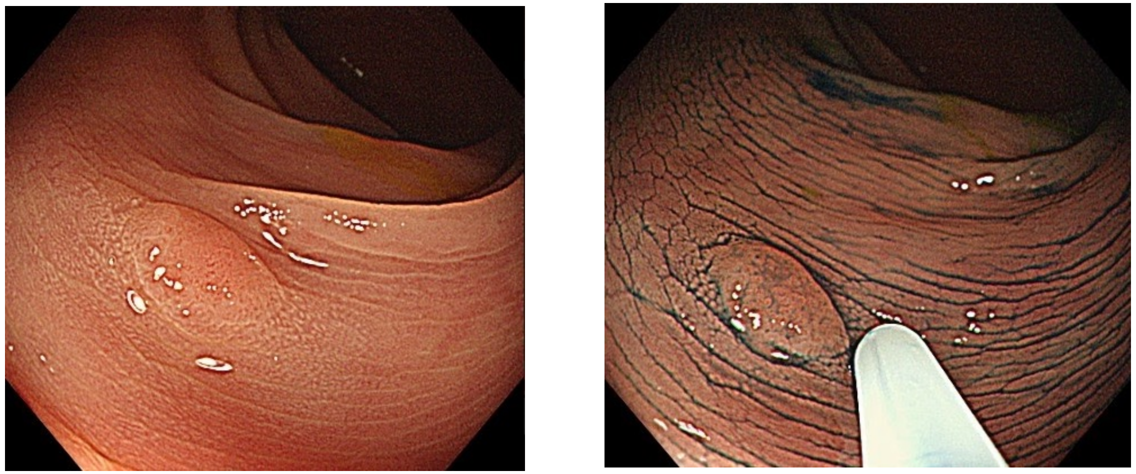

Diameter of jumbo biopsy forceps or snare (≒ 2.5 mm) ➡ **Higher than the device = Is**
Lower than the device = IIa

Department of Gastroenterology and Hepatology, Kyoto University Graduate School of Medicine

Rule ③

③ IIc vs “pseudo” IIc ▶ **The border of the depression (IIc) is clear !!**

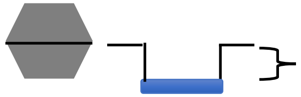 ＞1.2 mm ≒ Half the diameter of the forceps (jumbo) or snare

IIc
(depressed)

Difficult to judge the length !

The key is that the border between the depression and the surrounding mucosa is clear !

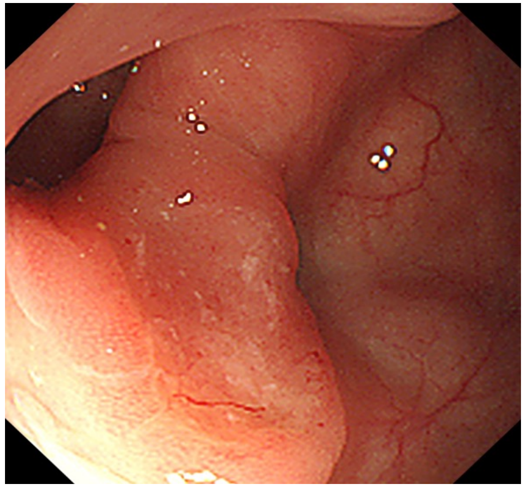

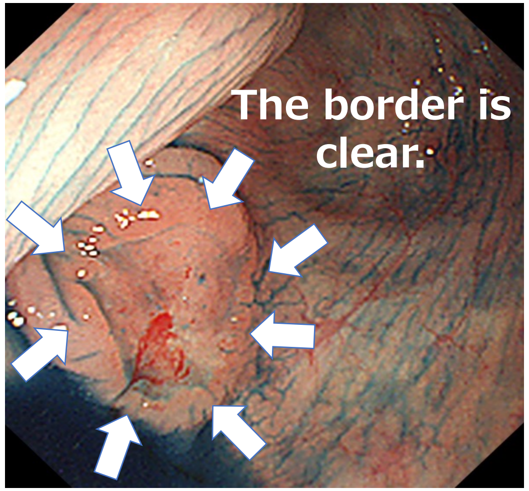

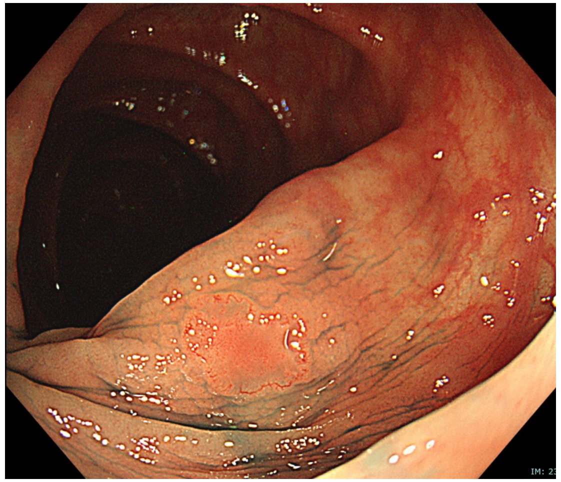

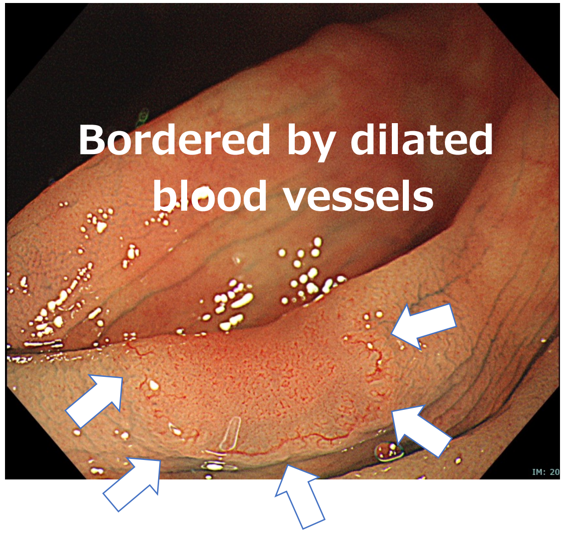

Rule ③

"Pseudo" IIc was previously called IIa+dep. ➡ **This is not real IIc.**

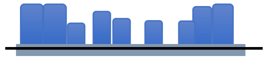

(the so-called)
IIa+dep

Although not classified as morphology, it has a depression with an unclear border in a flat elevated lesion.

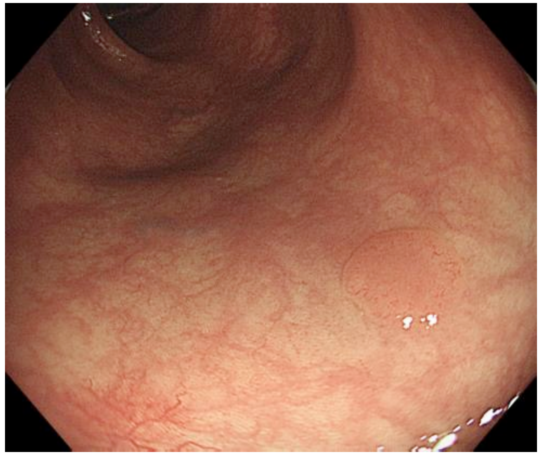

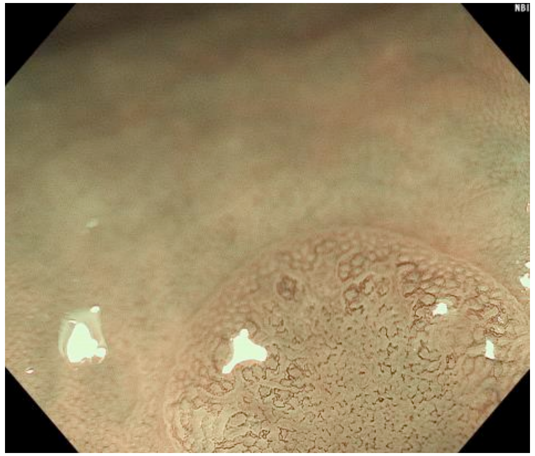

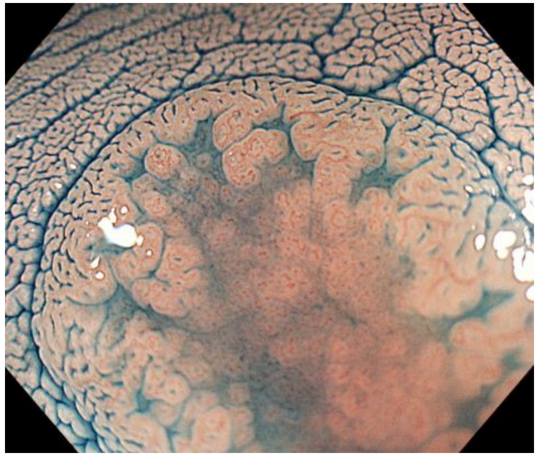

Salmon roe-like pits in depression with an unclear border

→ (Pathology)
Low grade adenoma

Rule ④

④ Mixed type ▶ First, describe the type with a larger area.

※ ‘Mixed type’ is used only when the lesion had two or more clearly different components.

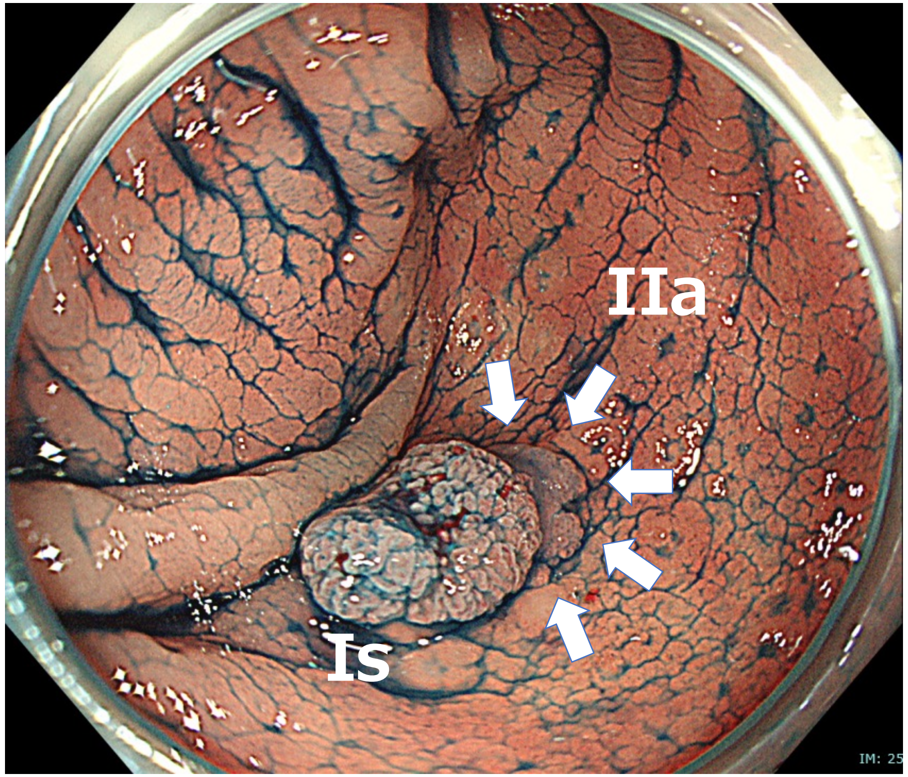

The lesion has two components.

The morphology with a larger area is・・・・
Is (left side)

The morphology with a smaller area is ・・・・
IIa (right side)

Therefore, the polyp morphology is ・・・・
Is (larger area) + IIa (smaller area)

Rule ④

Notes on complex types, including depressed types.

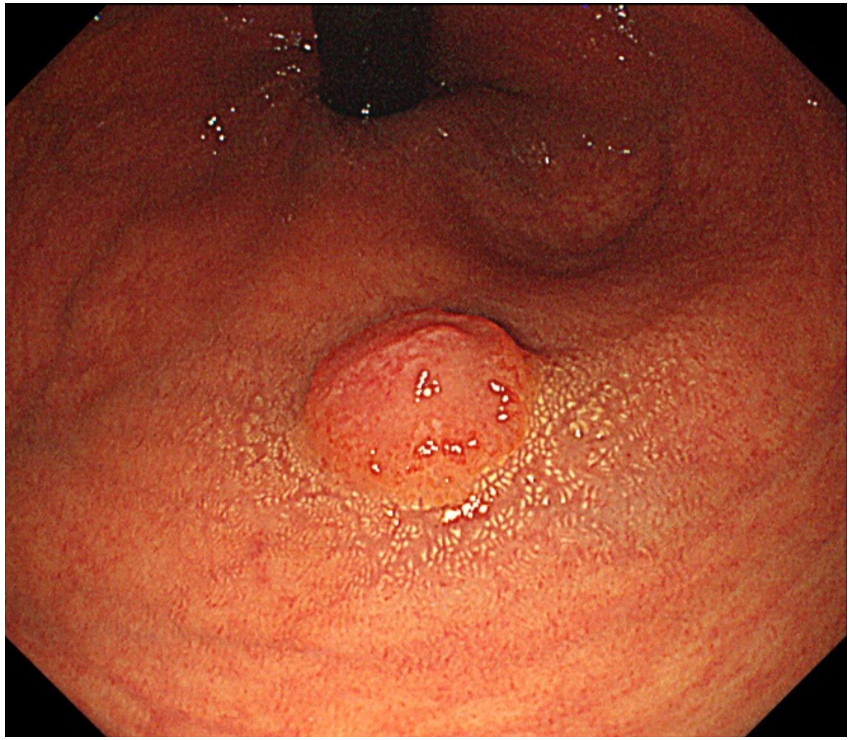

The morphology with a larger area is・・・・

IIc ✖

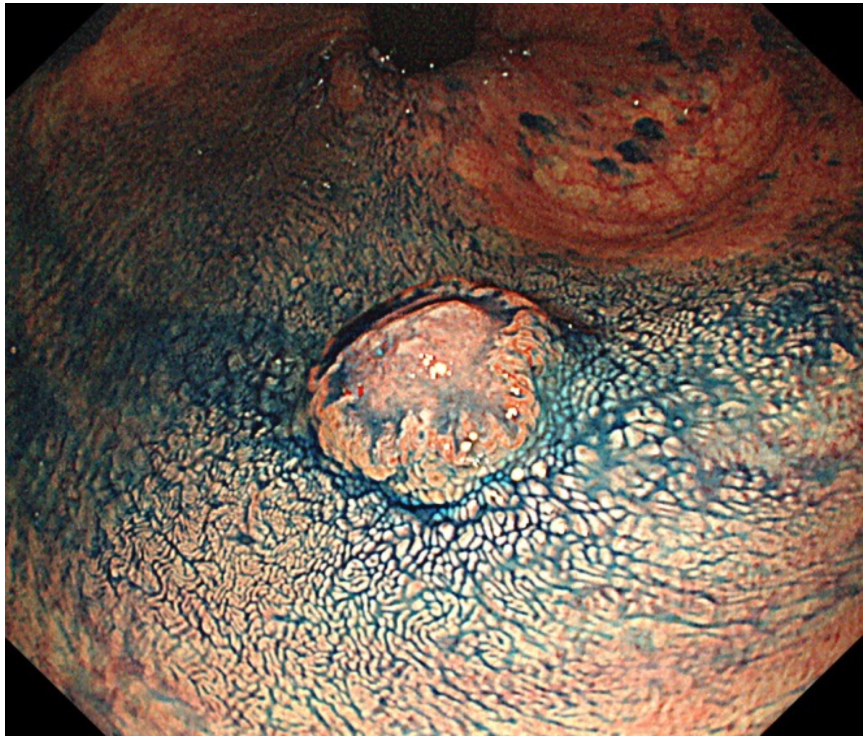

Rule ④

Notes on complex types, including depressed types.

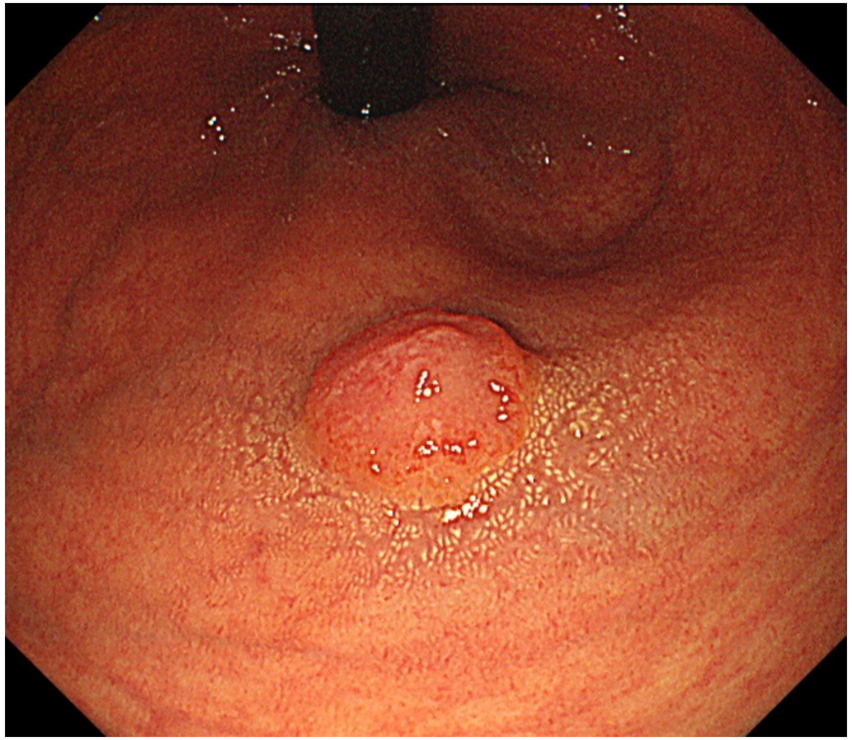

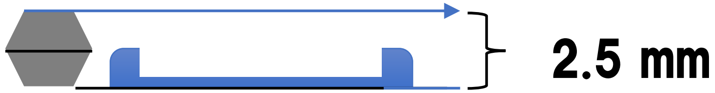

We should consider that the center of IIa showed a depressed area.

IIa + IIc

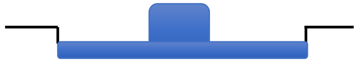

(Not IIc + IIa)

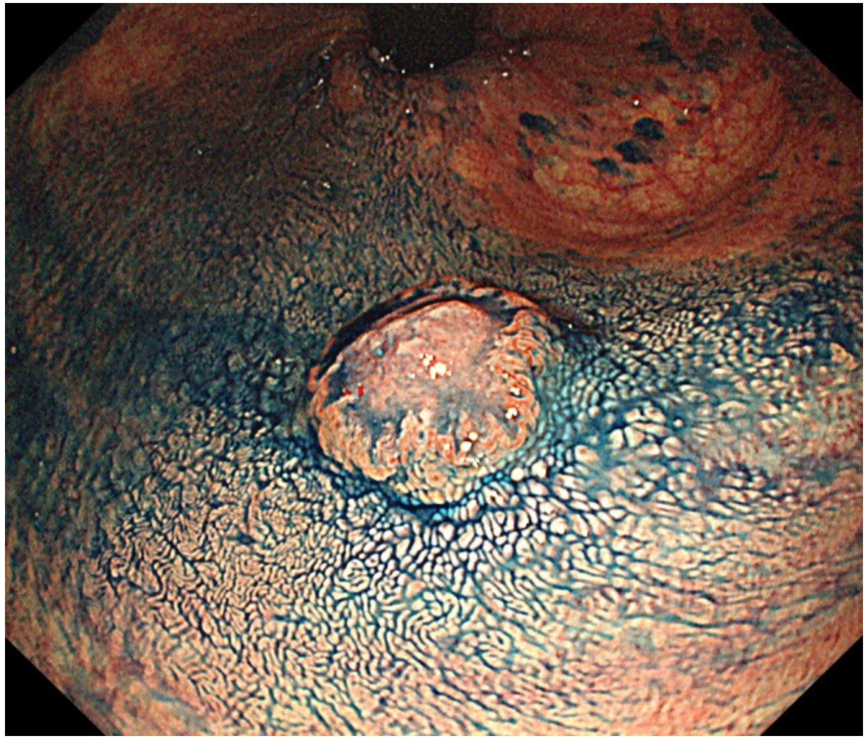

Rule ④

Notes on complex types, including depressed types.

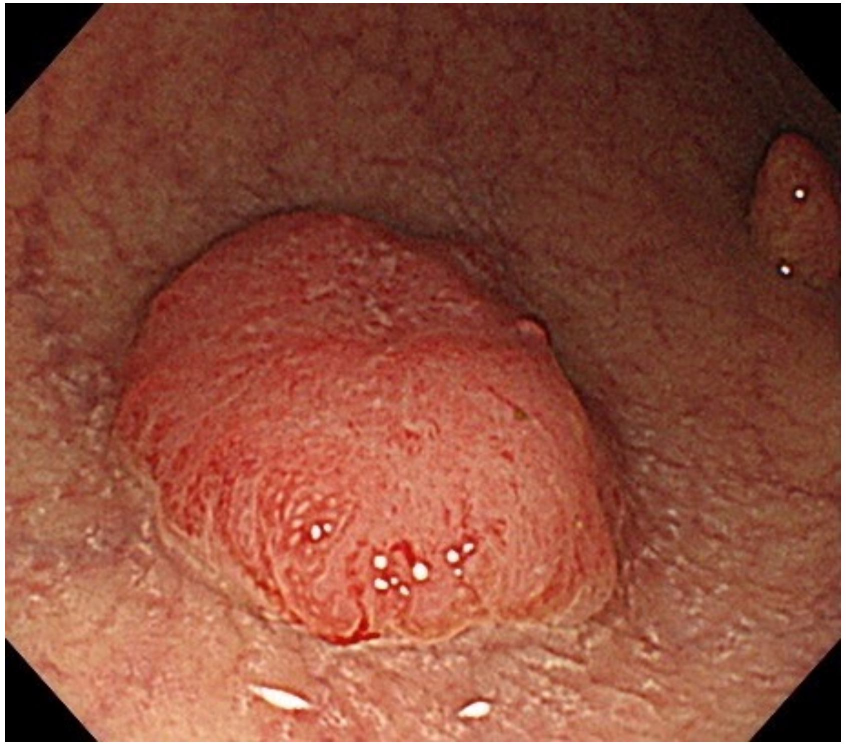

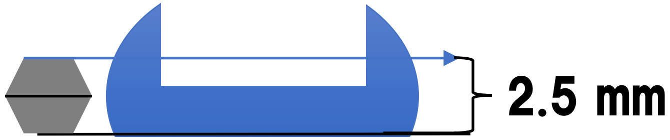

It should be considered that the surface of Is has a depressed area.

Is + IIc

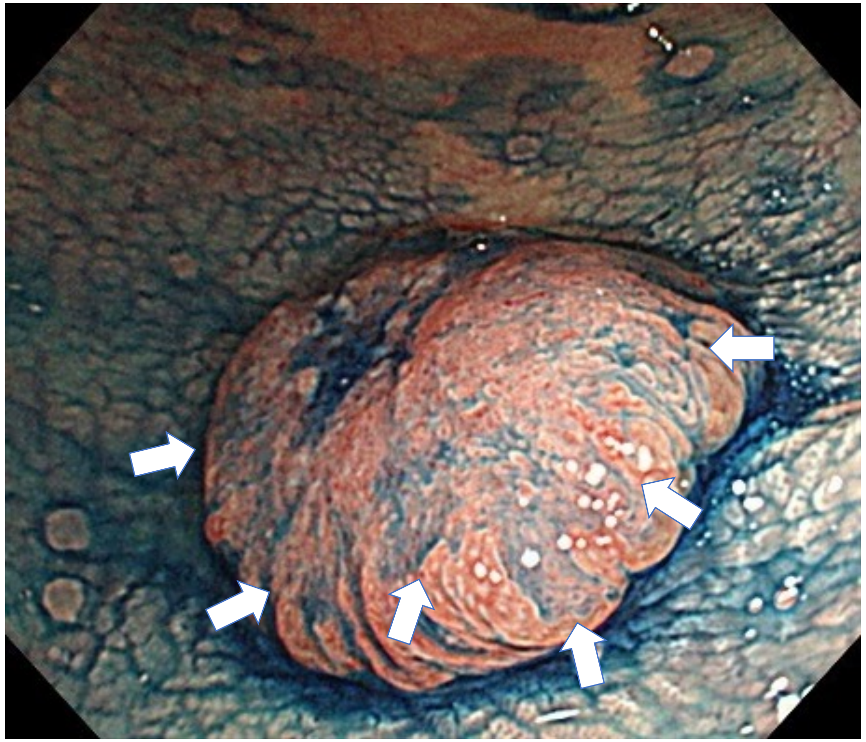

CONTENTS

1. Introduction – classification of morphology -

2. Rule for colorectal polyp morphology assessment

3. Exercises

Let’s now review LST subclassification!

Laterally spreading tumor (LST) is not officially a morphological type, but it is important to learn about LST!

LST (nickname for flat elevated lesions ≥10 mm)

Rule A

- Granular type: **LST-G**
  - **Rule B**
    - Homogenous (Homo)
    - Nodular Mixed (MIX)
- Non-Granular type: **LST-NG**
  - **Rule C**
    - Flat elevated（FE)
    - Pseudo-depressed（PD)

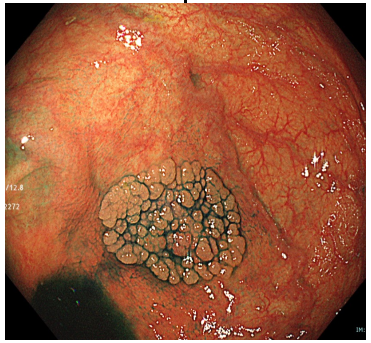

Homogenous (Homo)

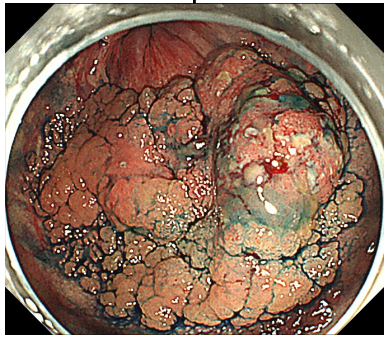

Nodular Mixed (MIX)

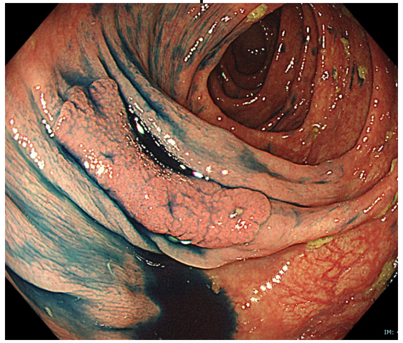

Flat elevated（FE)

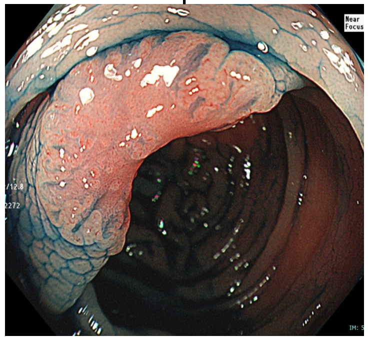

Pseudo-depressed（PD)

Extra Rule A

A. LST-G vs LST-NG ▶ Granular or Grooved ?

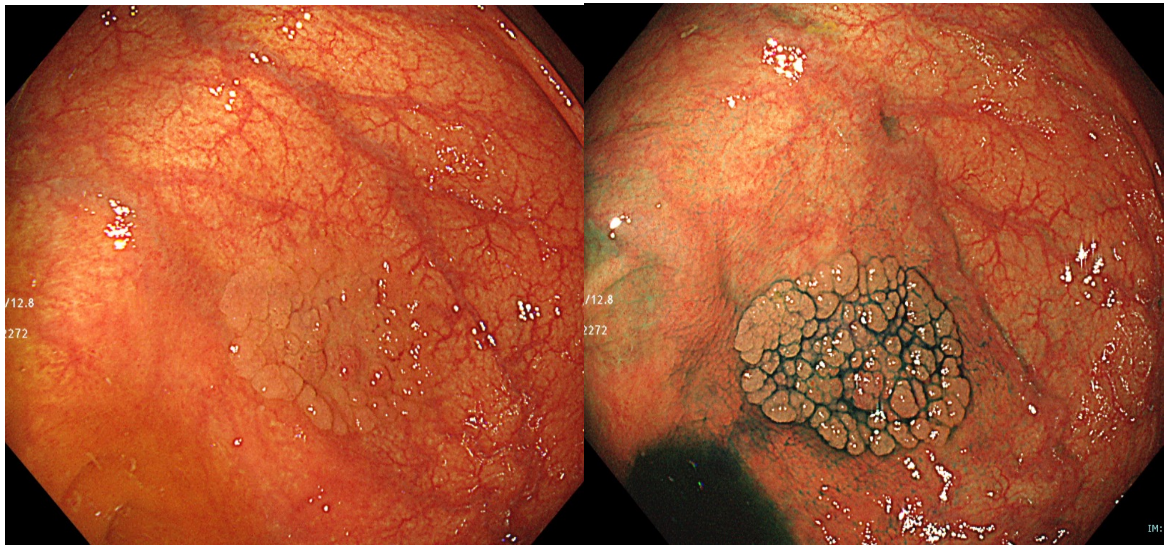

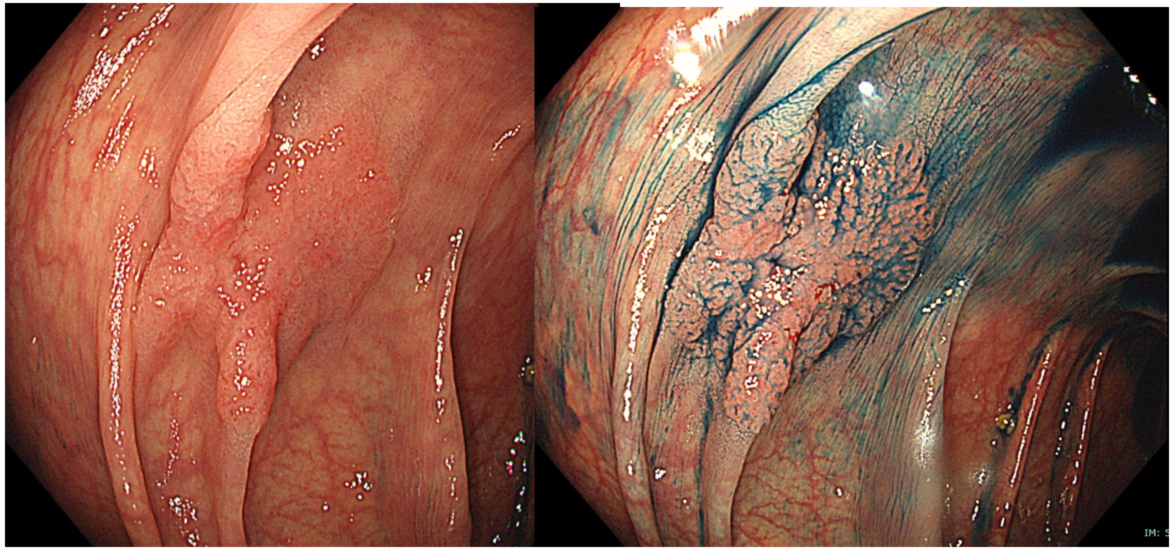

Granular (LST-G) | **Grooved (LST-NG)**

YES | NO

Can you circle each granule using ○ ?

Department of Gastroenterology and Hepatology, Kyoto University Graduate School of Medicine

Extra Rule B

B. LST-G (homo) vs (MIX) ▶ Are the granules uniform ?

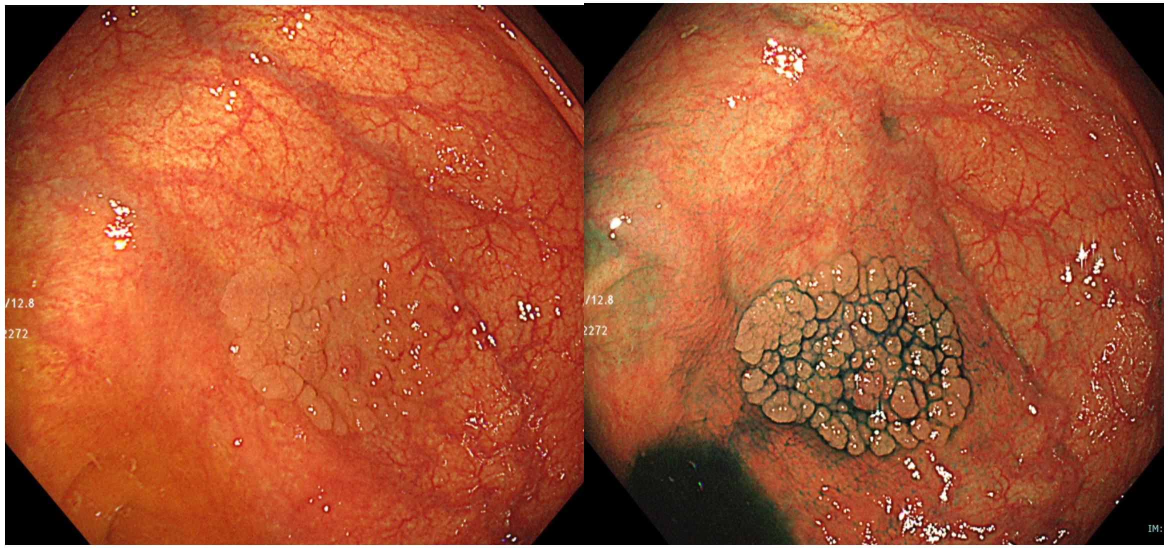

Uniform (Homo)

When all the granules are ≤5 mm, the lesion is easily diagnosed as homogeneous.

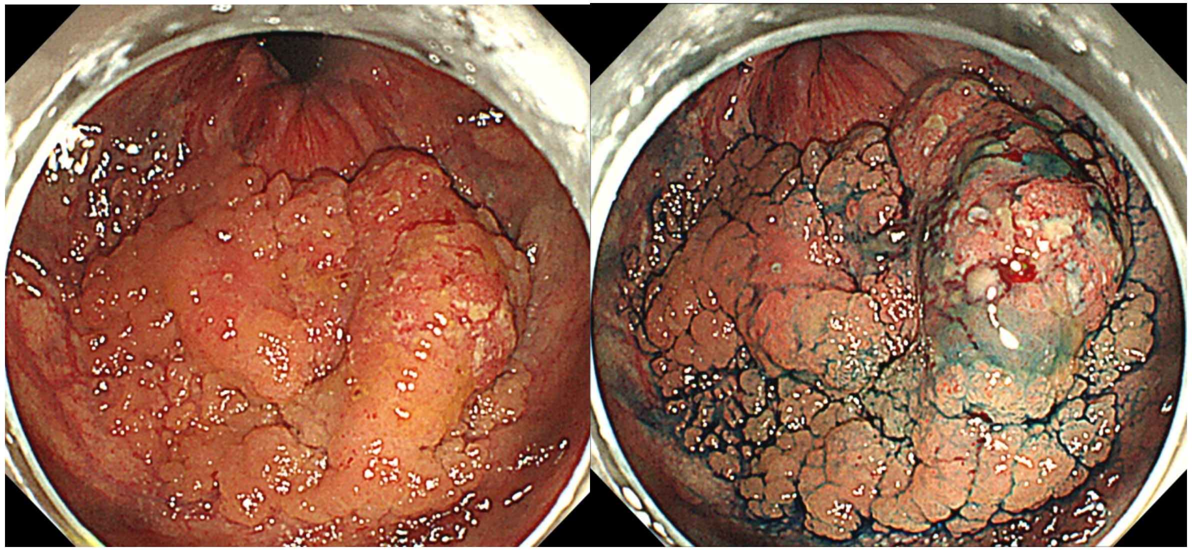

Not uniform (Mix)

When coarse nodules are ≥10 mm, the lesion is easily diagnosed as heterogeneous.

Are the granules really uniform?

YES → Uniform (Homo)

NO → Not uniform (Mix)

Extra Rule C

C. LST-NG (FE) vs (PD)

The lesion has a pseudo-podium-like appearance?

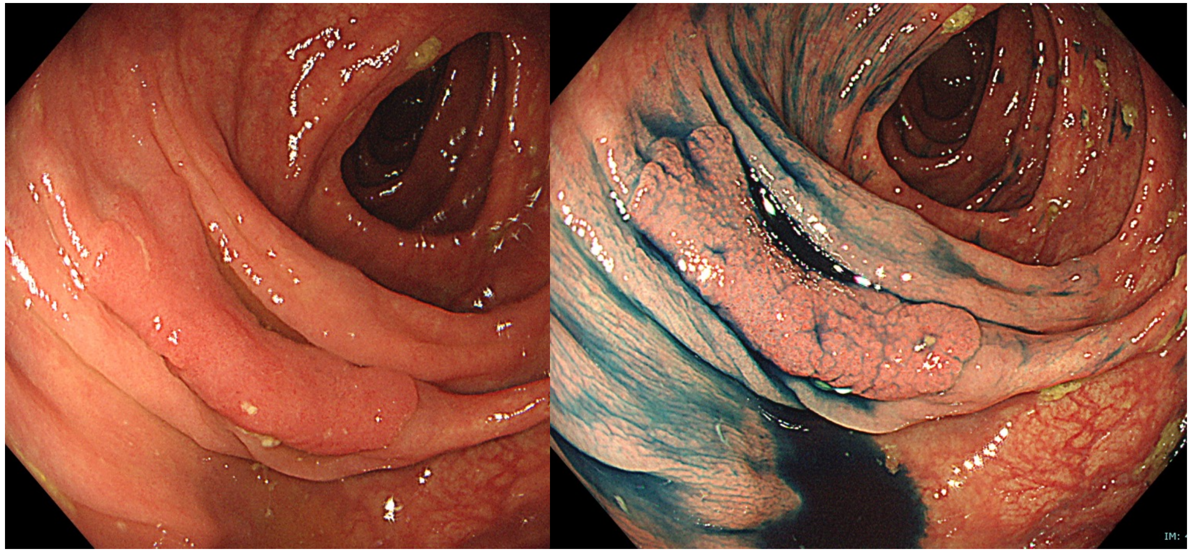

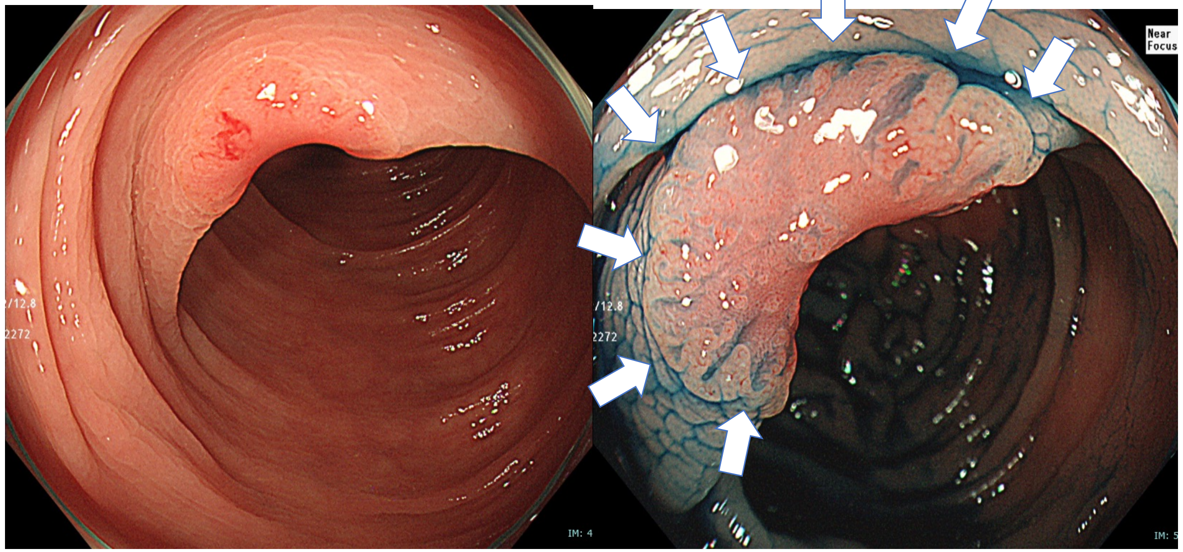

LST-NG(FE)

LST-NG(PD)

NO

YES

Let’s remember pseudo-podium-like findings in the next slide!

Does the lesion (slightly depressed in the center) have a pseudo-podium-like finding at the edge?

Extra Rule C

Pseudo-podium-like appearance

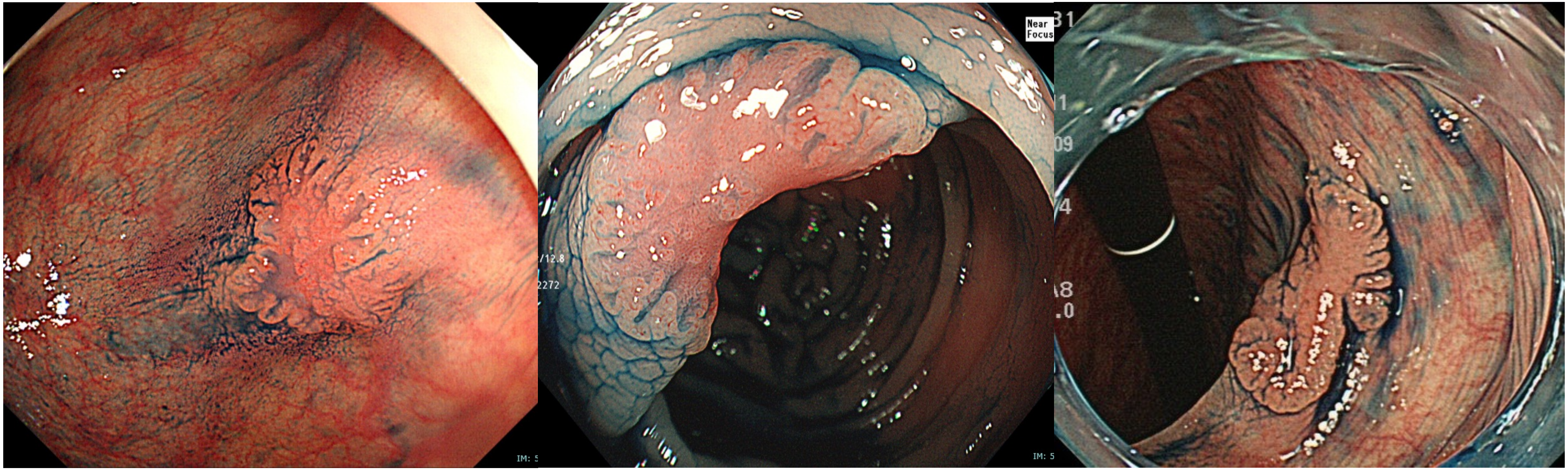

The amoeboid-like appearance (spreading into the surroundings at the edge)

Take Home Message (Morphology)

① Ip vs Is ▶ Ip has an obvious stalk !

② Is vs IIa ▶ Refer to a device diameter of 2.5mm !!

③ IIc vs “pseudo” IIc ▶ The border of the depression (IIc) is clear !!

④ Mixed type ▶ First, describe the type with a larger area.

Take Home Message (LST)

A. LST-G vs NG ▶ Granular or Grooved ?

B. LST-G(Homo)vs(Mix) ▶ Are the granules uniform ?

C. LST-NG(FE)vs(PD) ▶ The lesion has pseudo-podium-like appearance?

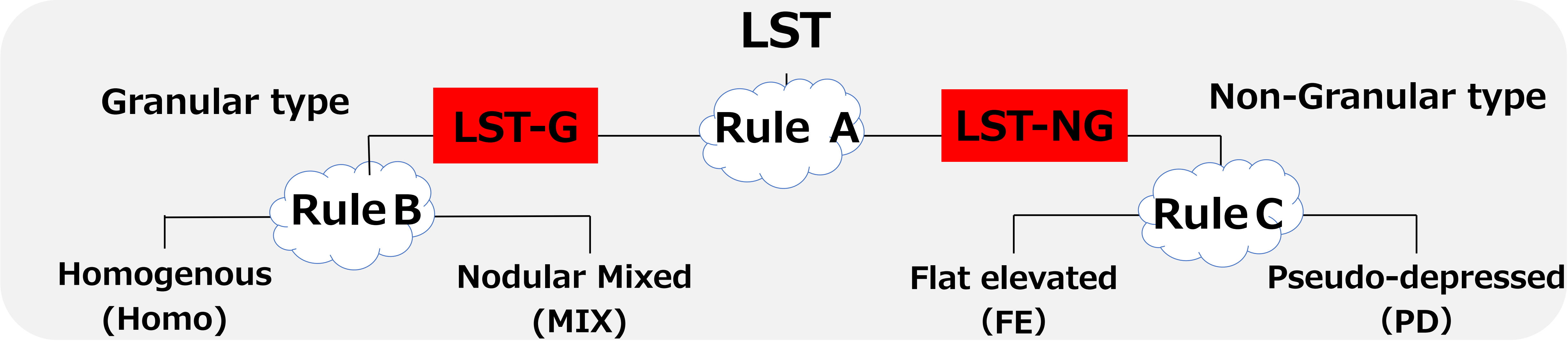

Question ①

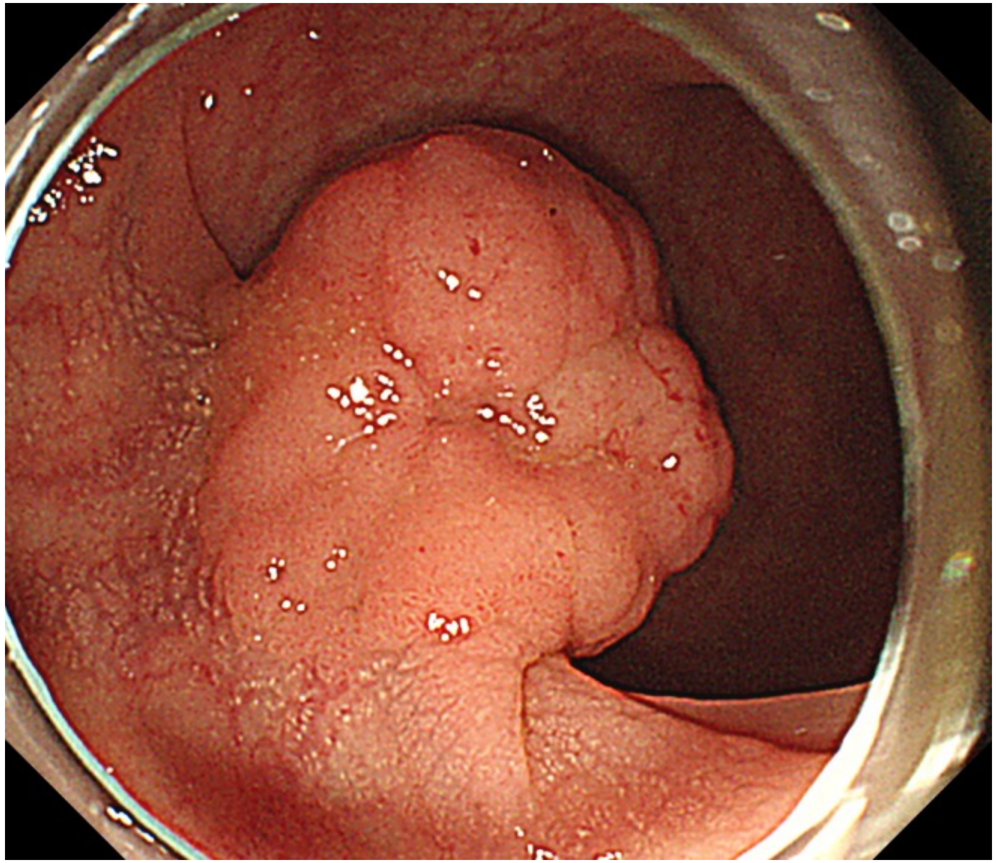

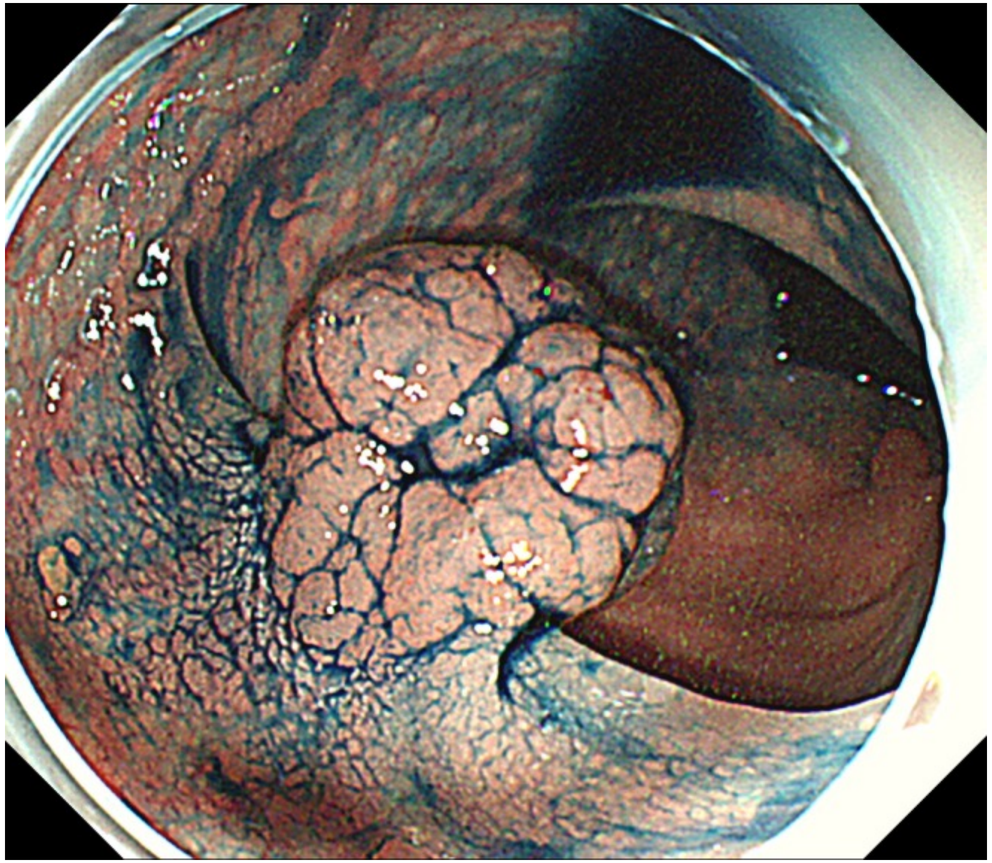

Q.　What is the morphological classification of this polyp ?

Question ①

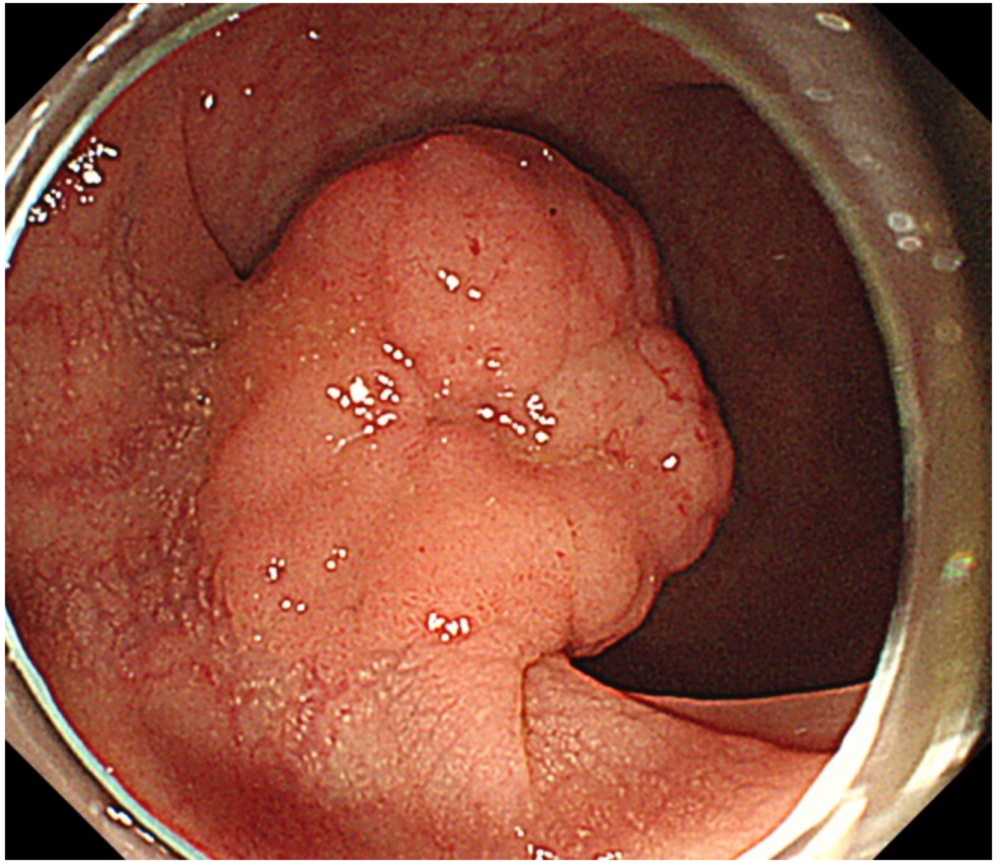

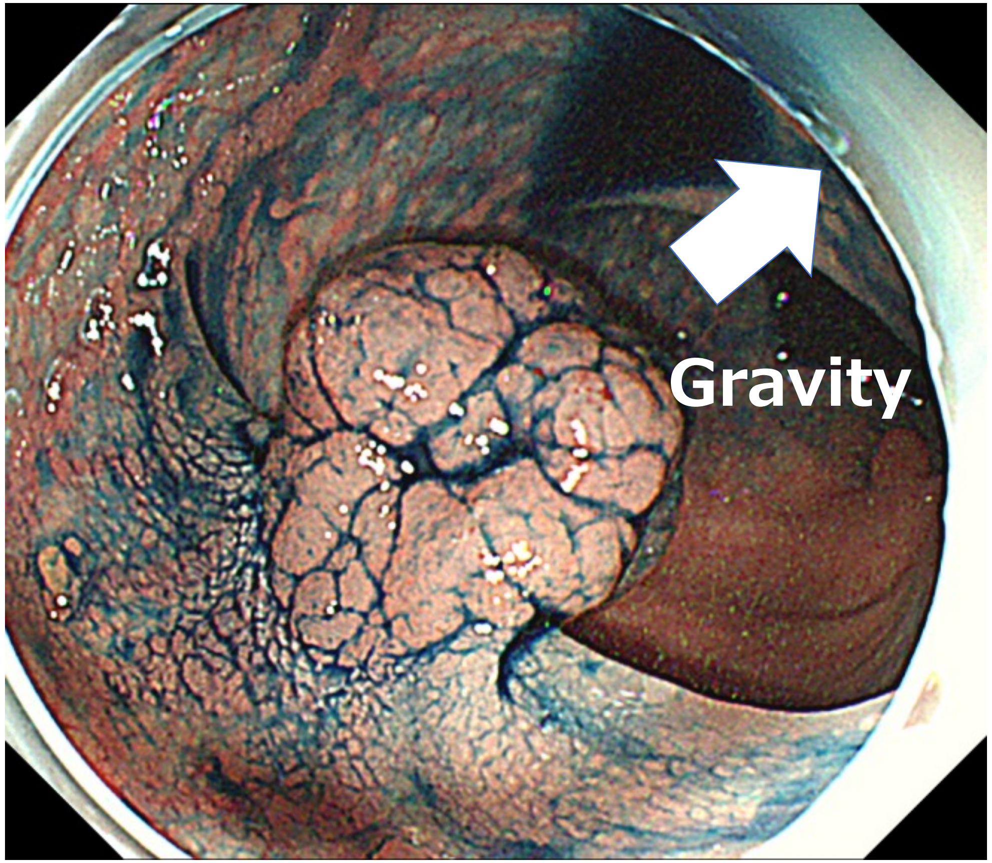

A.　Is　(No obvious stalk. Just hanging down due to gravity.)

Question ②

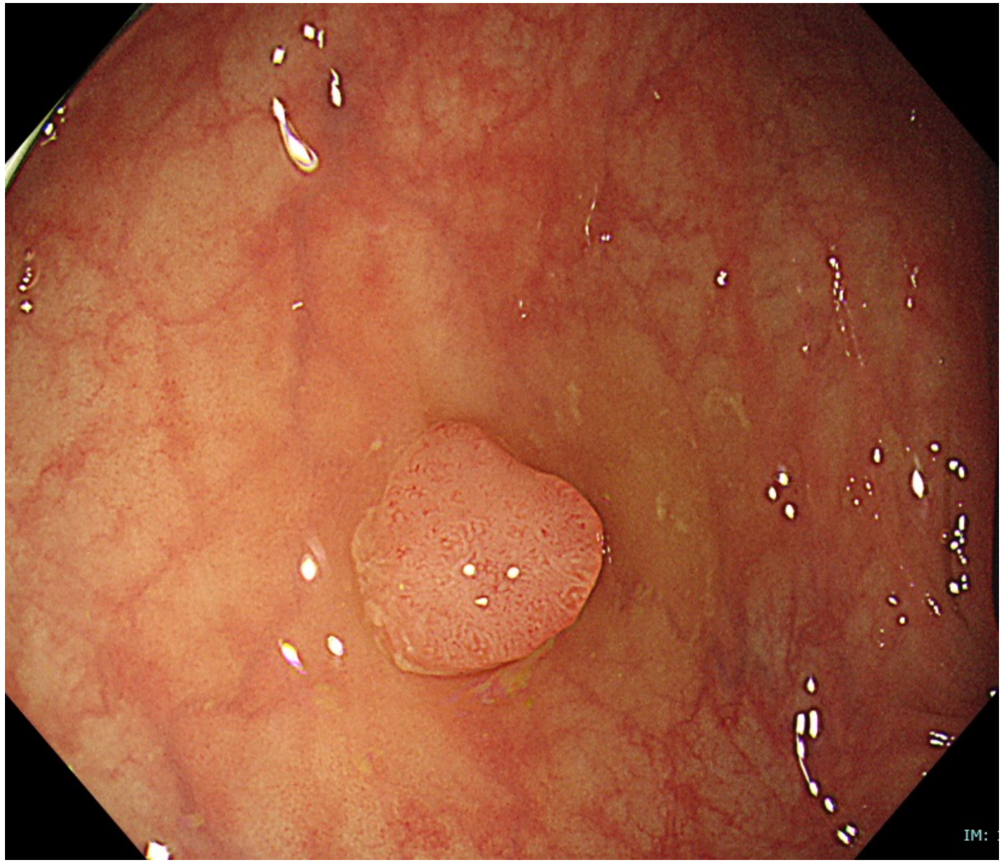

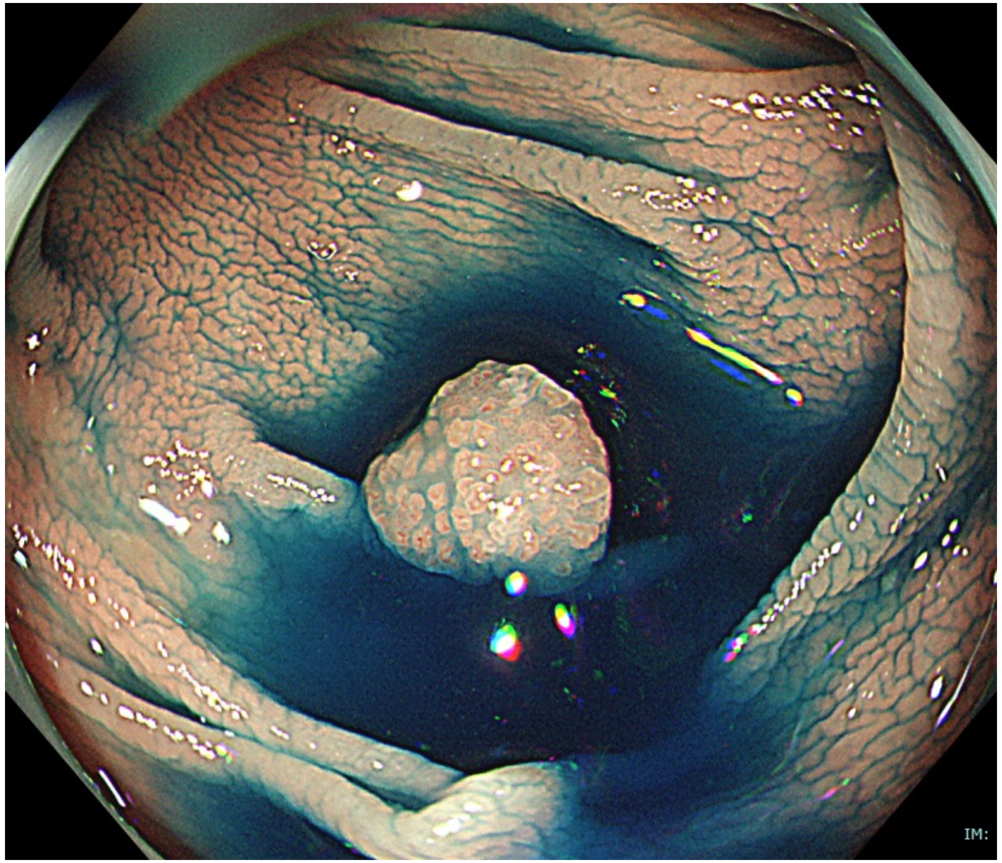

Q. What is the morphological classification of this polyp ?

Question ②

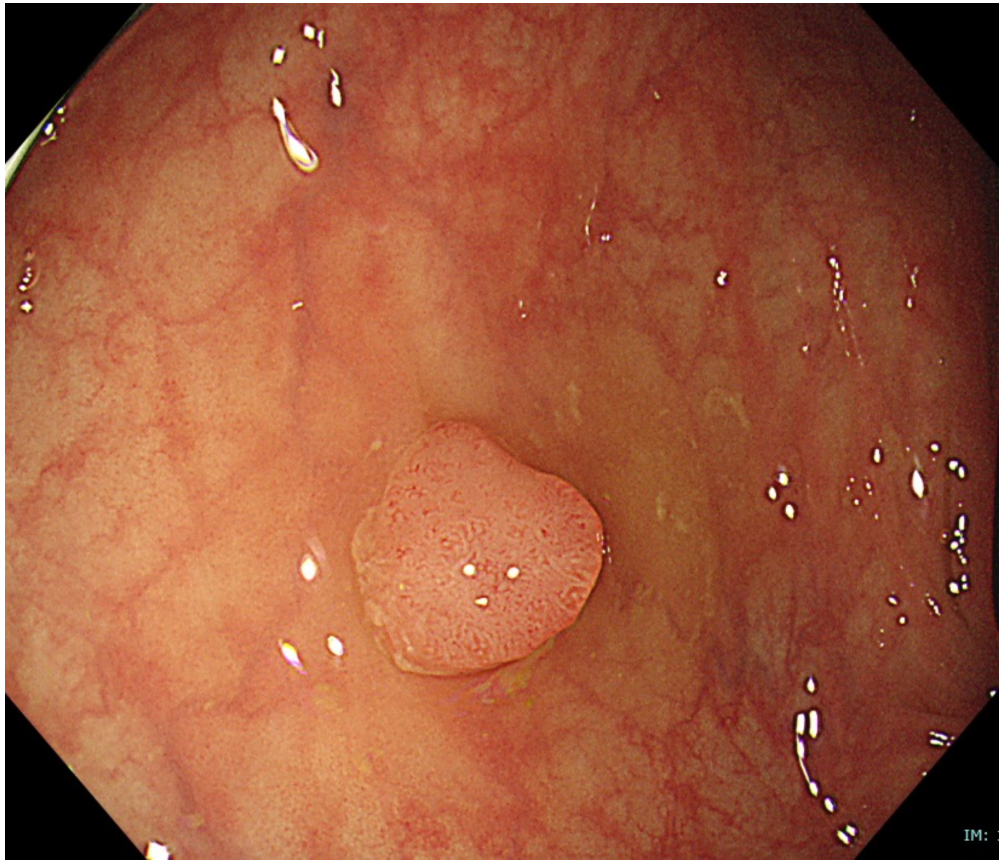

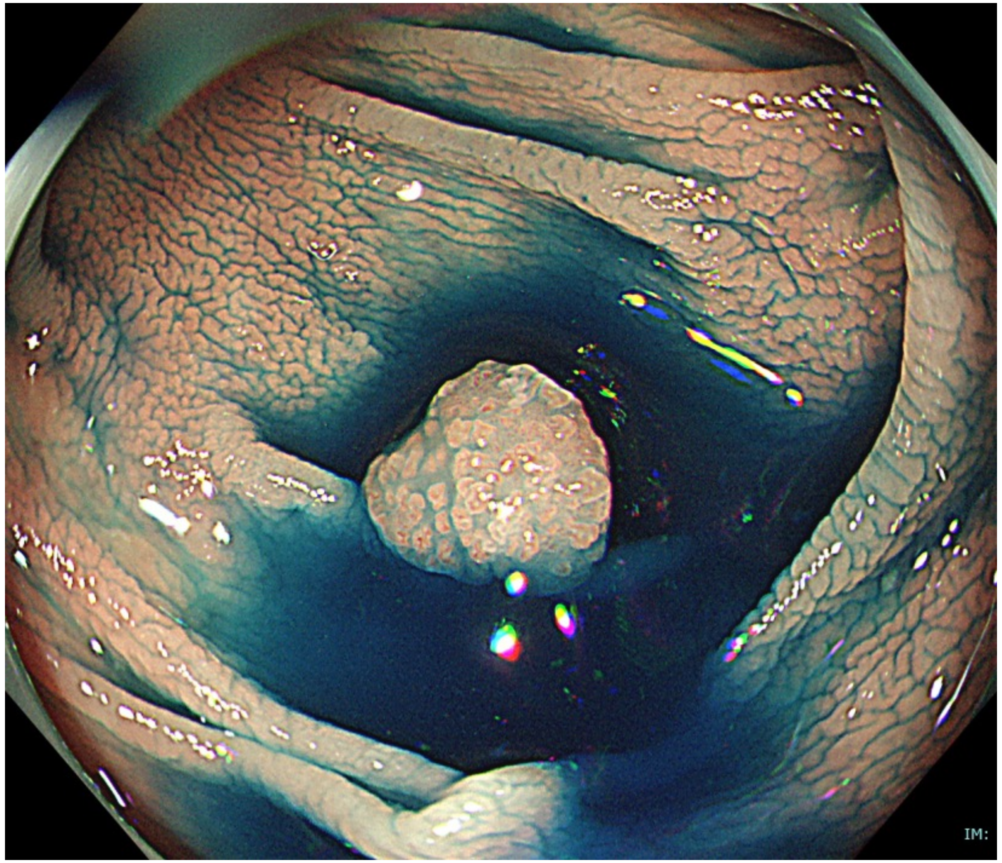

A. Is (The polyp is higher than the diameter of the forceps (jumbo) or snare)

Question ③

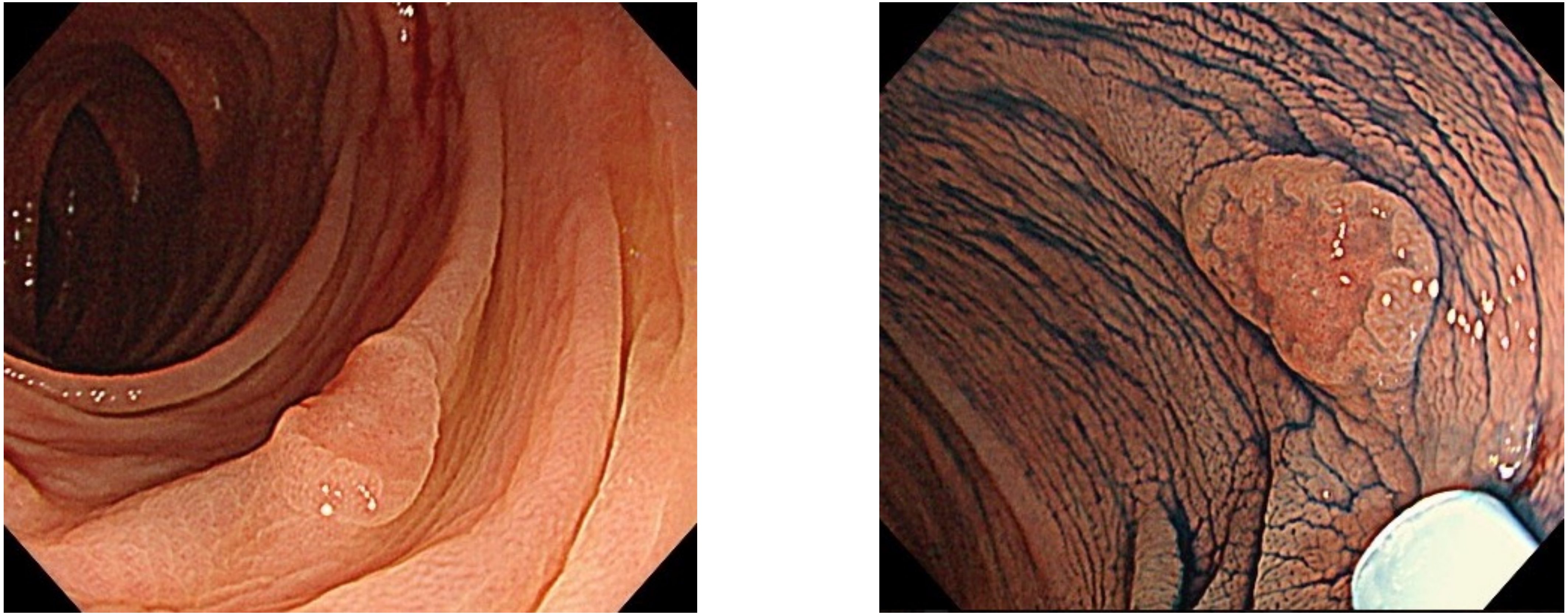

Q.　What is the morphological classification of this polyp ?

Question ③

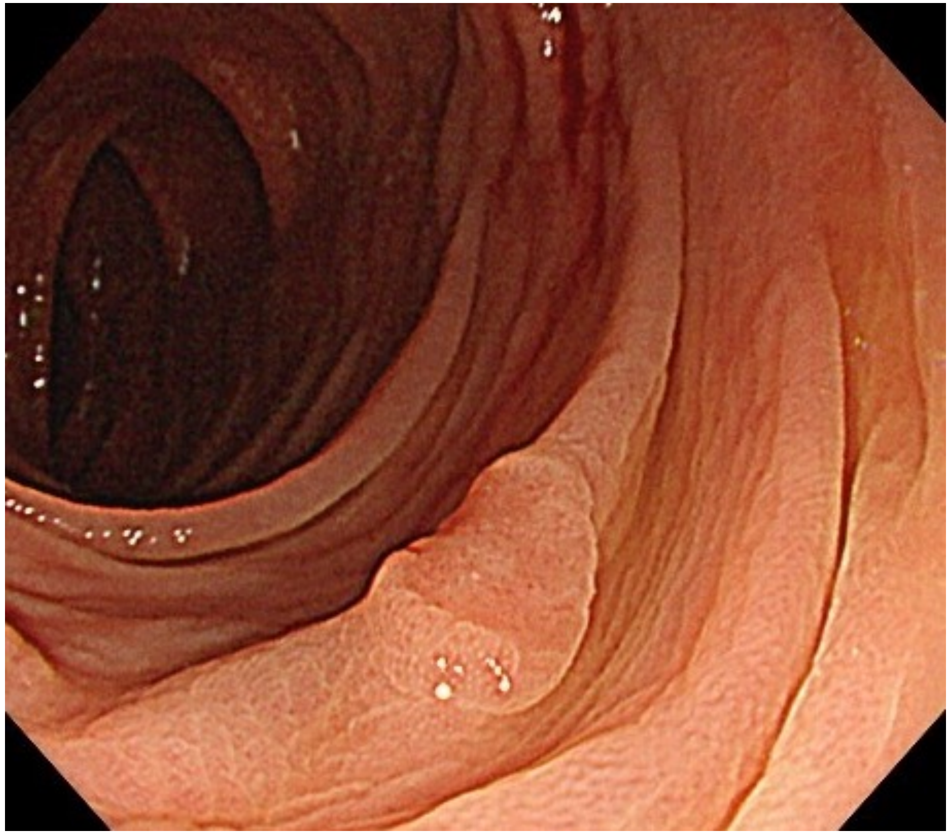

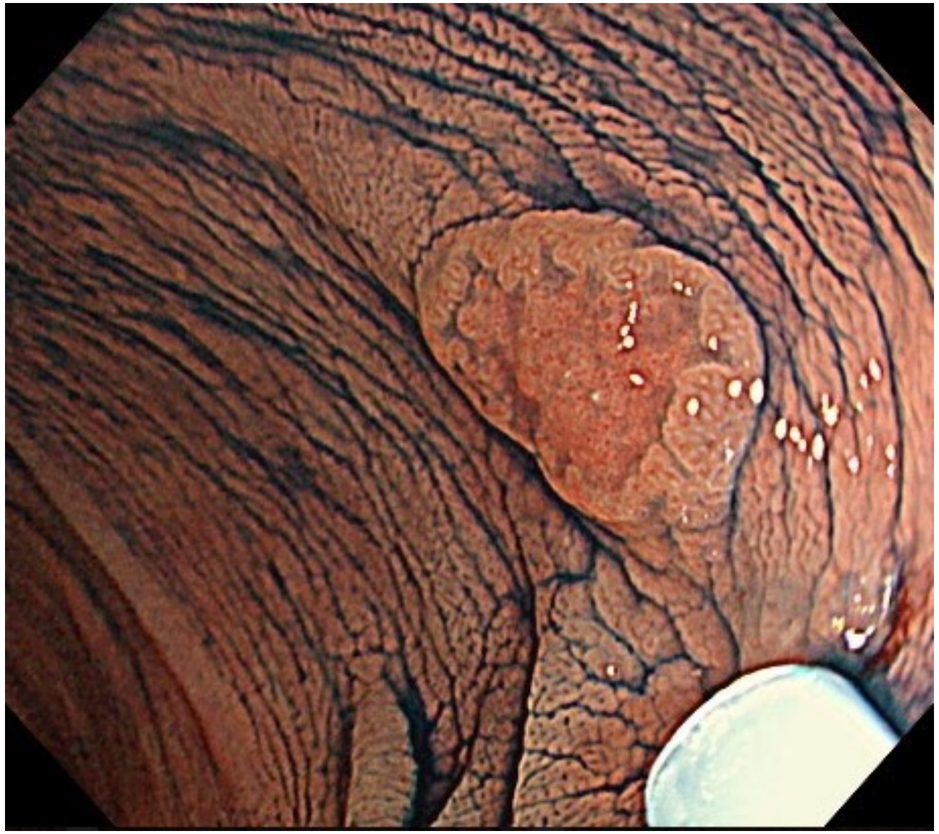

A.　IIa　**(The flat elevated polyp appears to have a depressed area, but the border is unclear.)**

Question ④

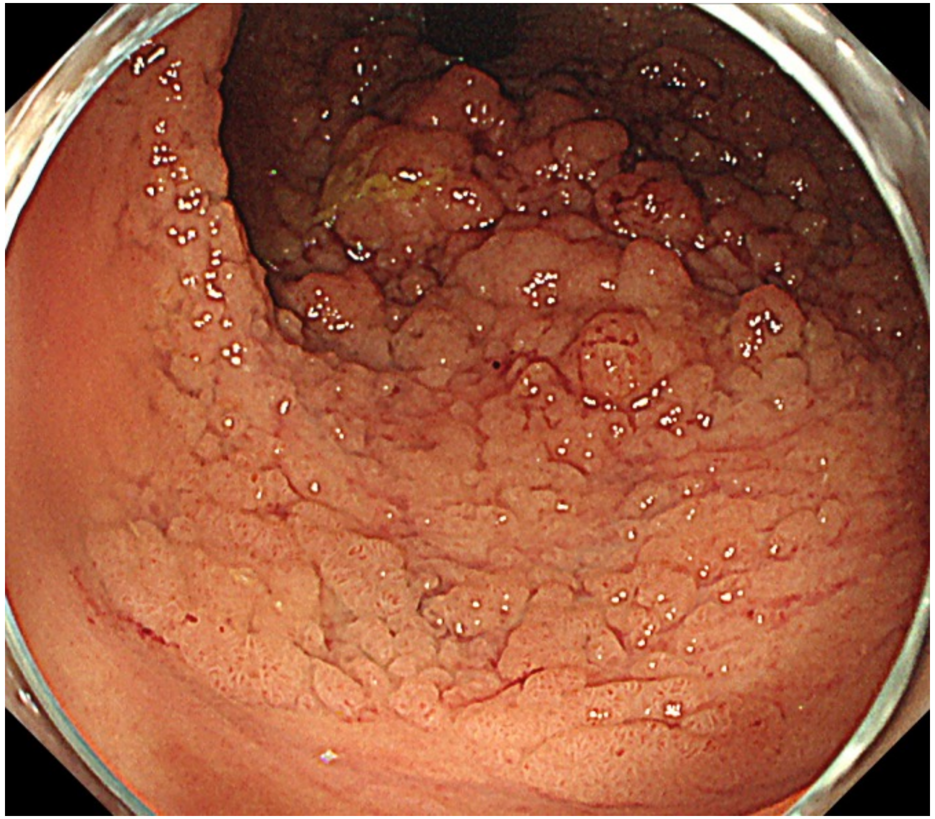

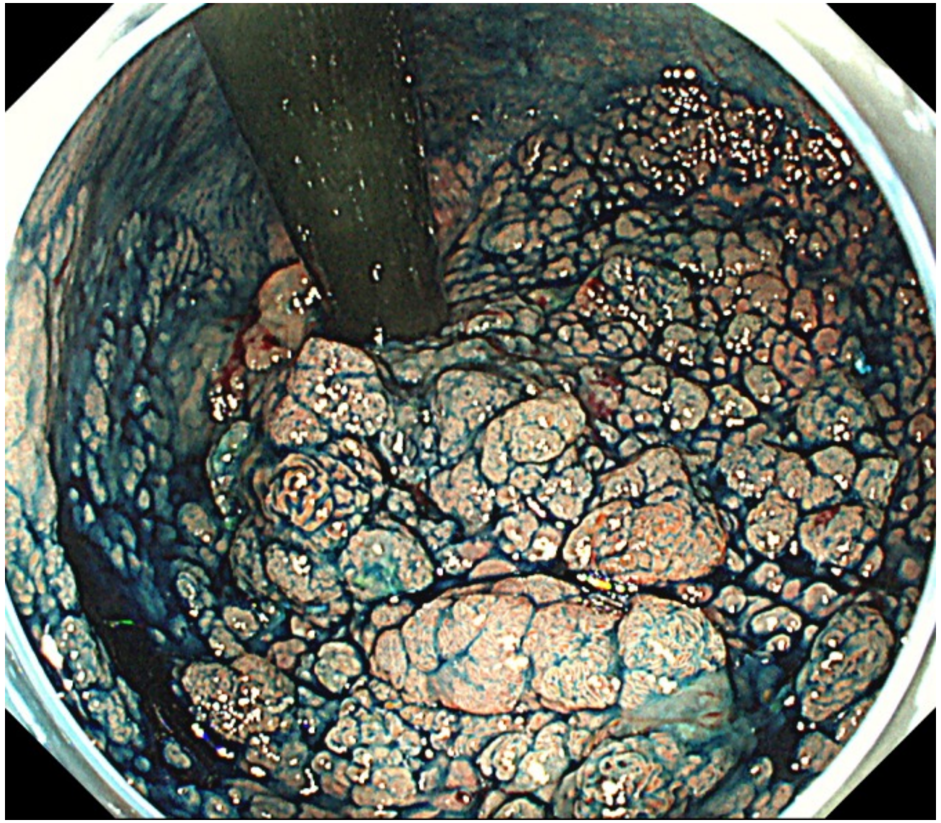

Q.　What is the morphological classification of this polyp ?

Question ④

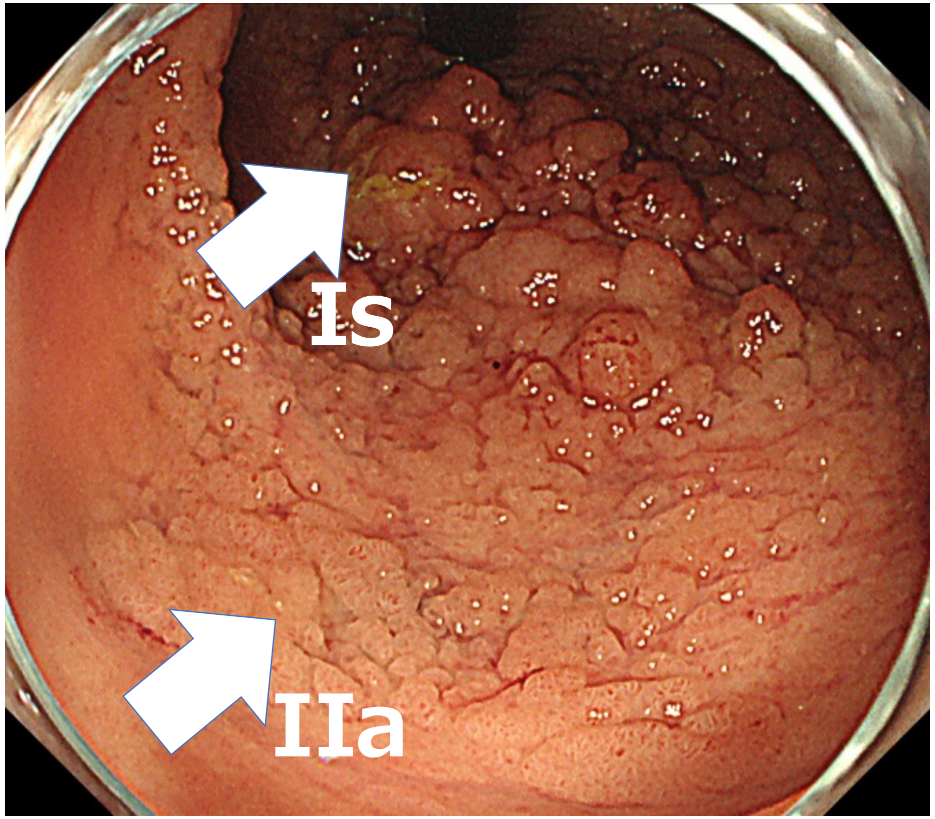

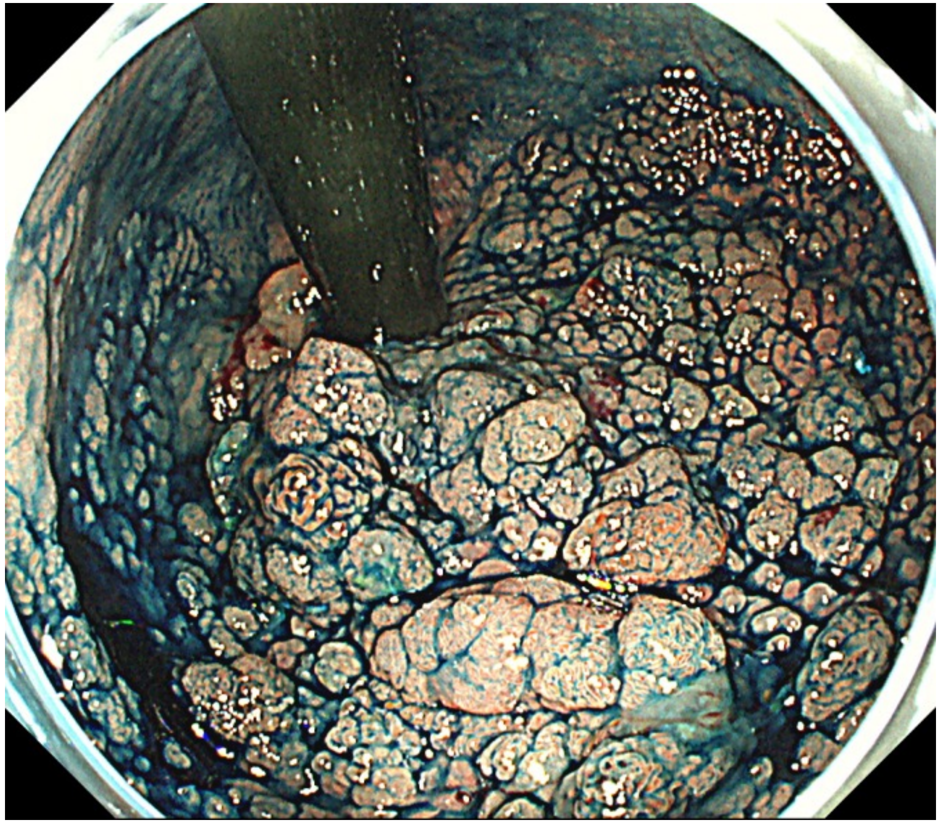

A. **IIa + Is** (The larger area is IIa. The smaller area is Is.)

Question ⑤

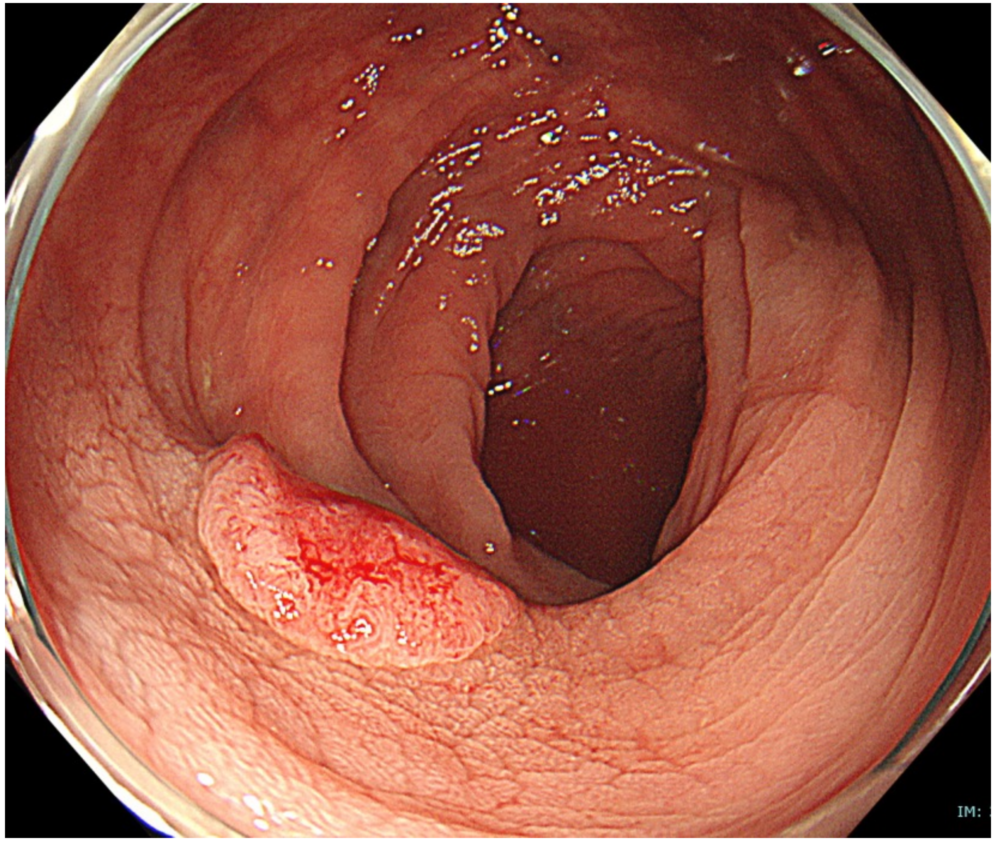

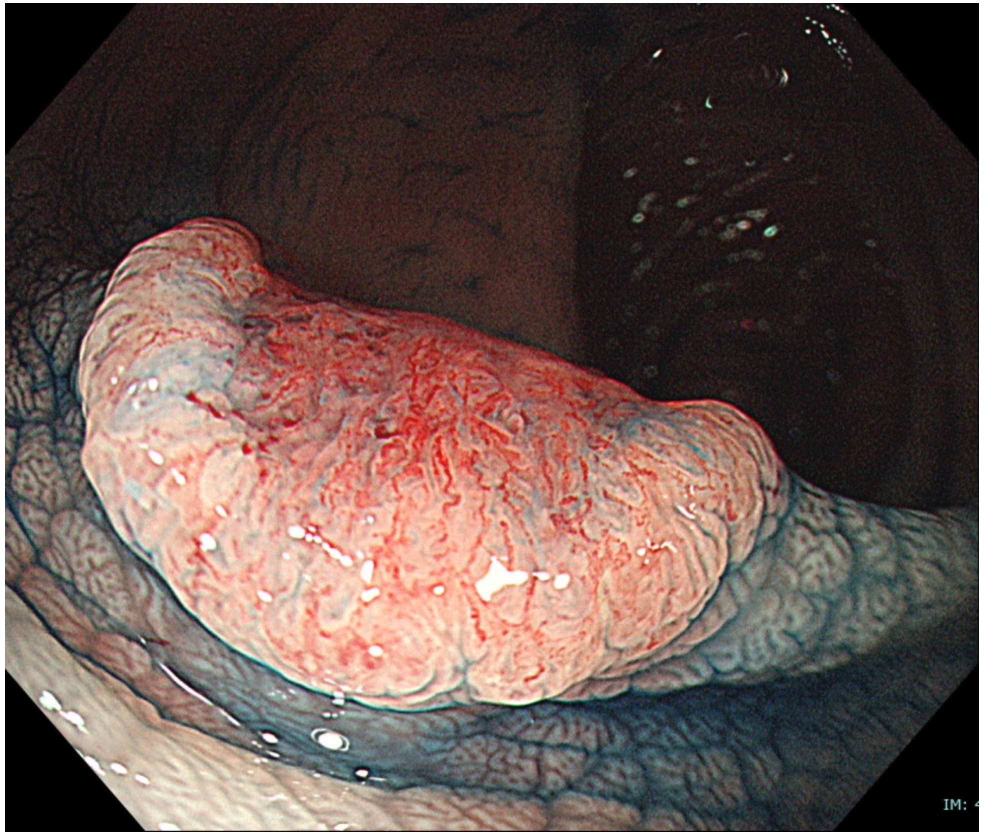

Q.　What is the morphological classification of this polyp ?

Question ⑤

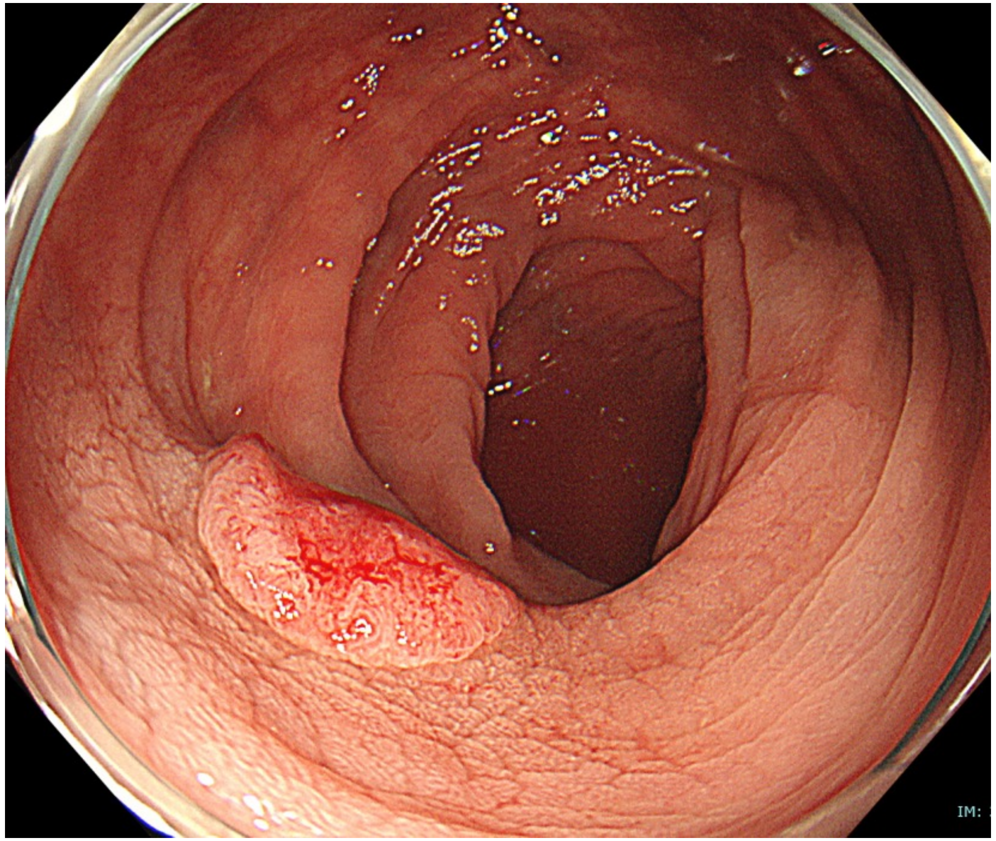

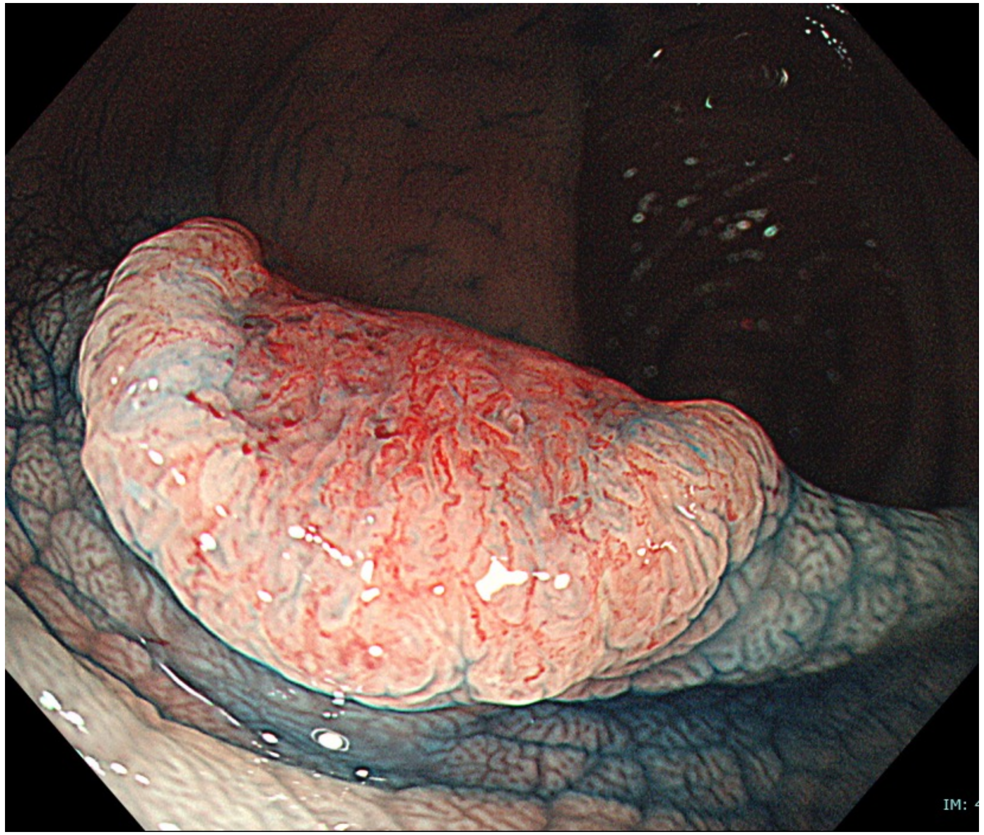

A.　IIa + IIc　(Not IIc + IIa)

Question ⑥

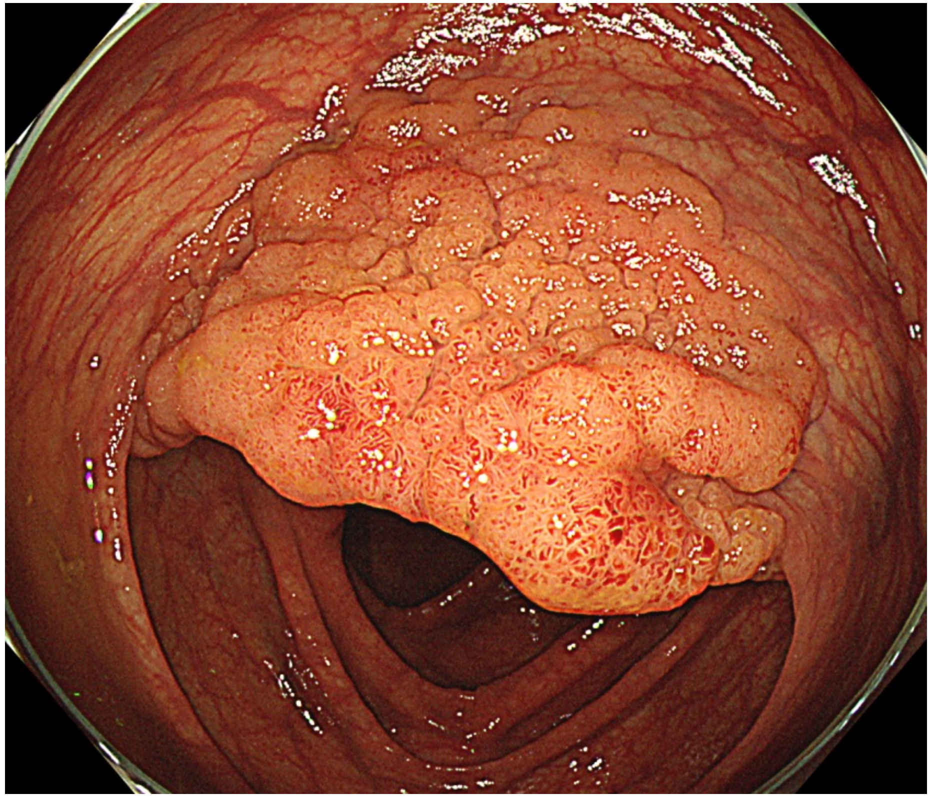

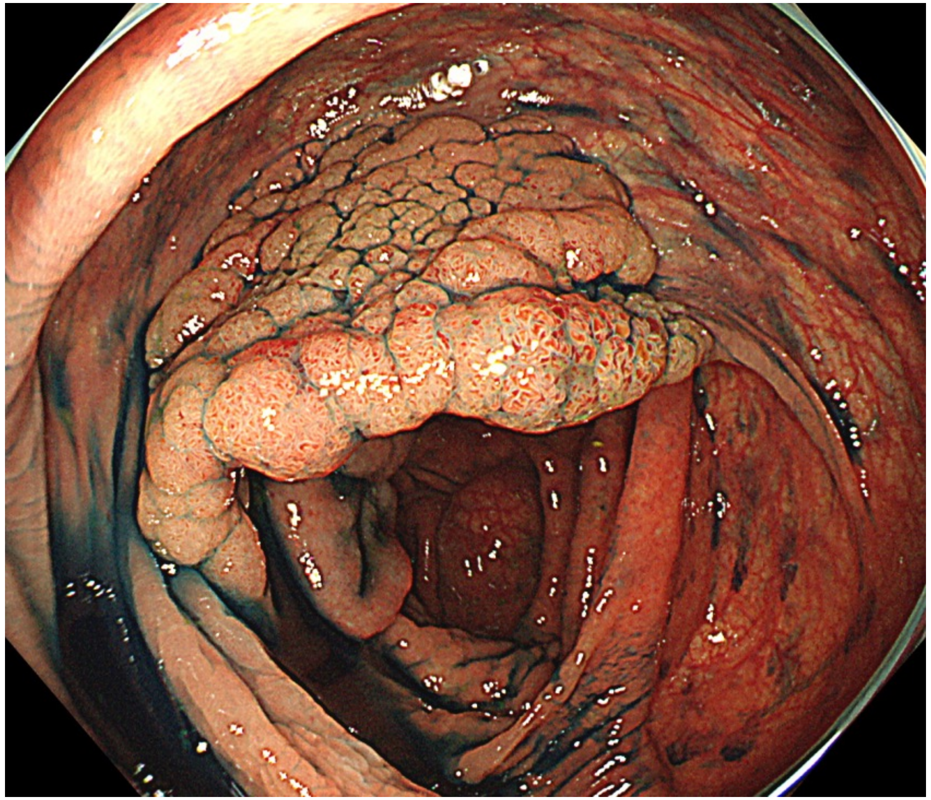

Q.　What is the morphological subclassification of this LST ?

Question ⑥

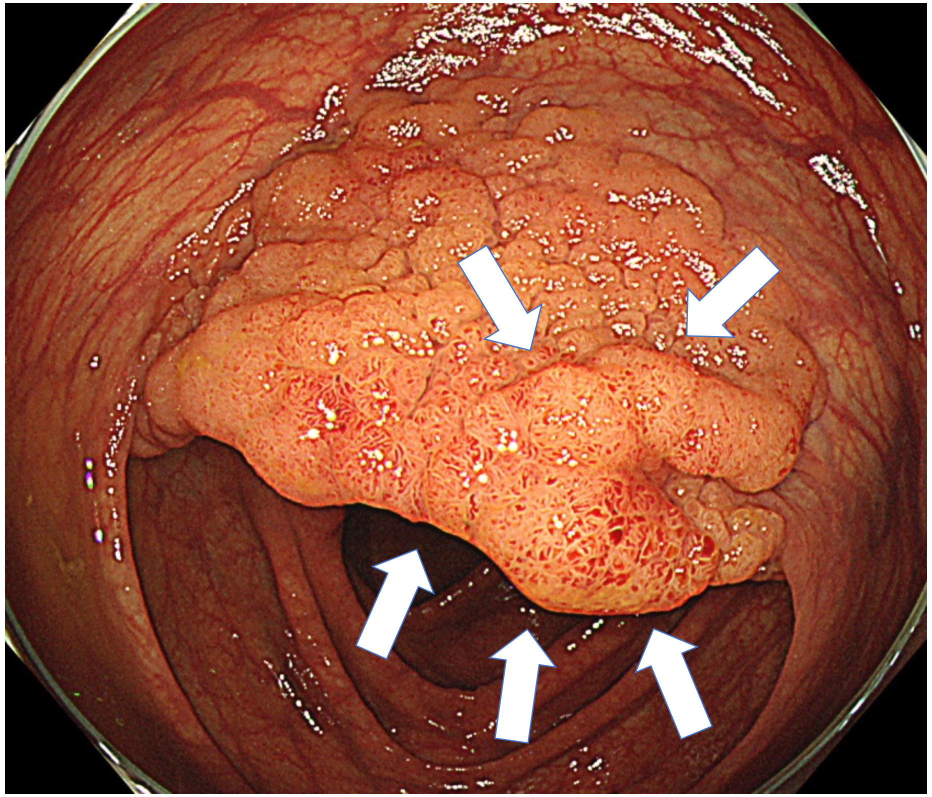

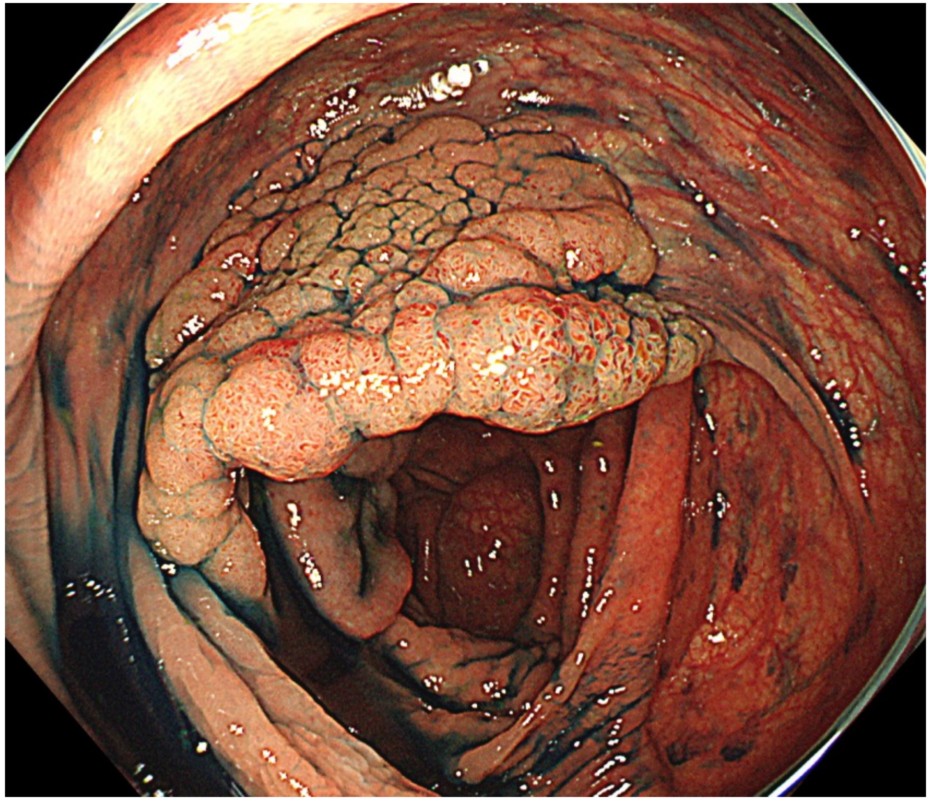

A. LST-G (MIX) (The lesion has granules that are not uniform.)

Question ⑦

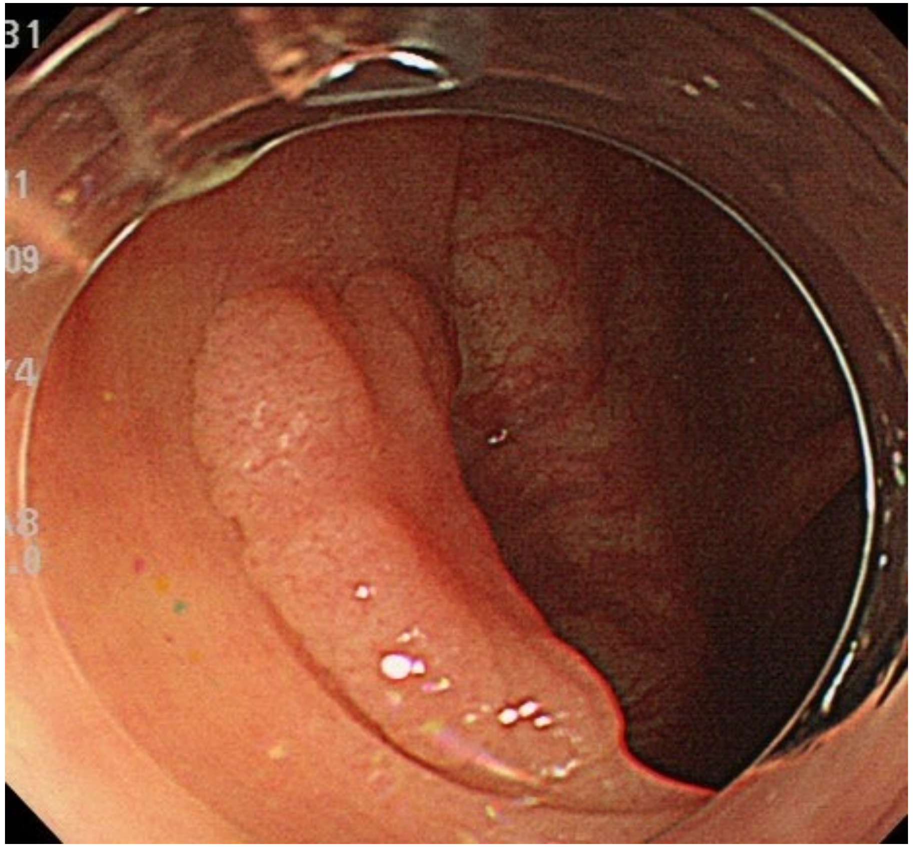

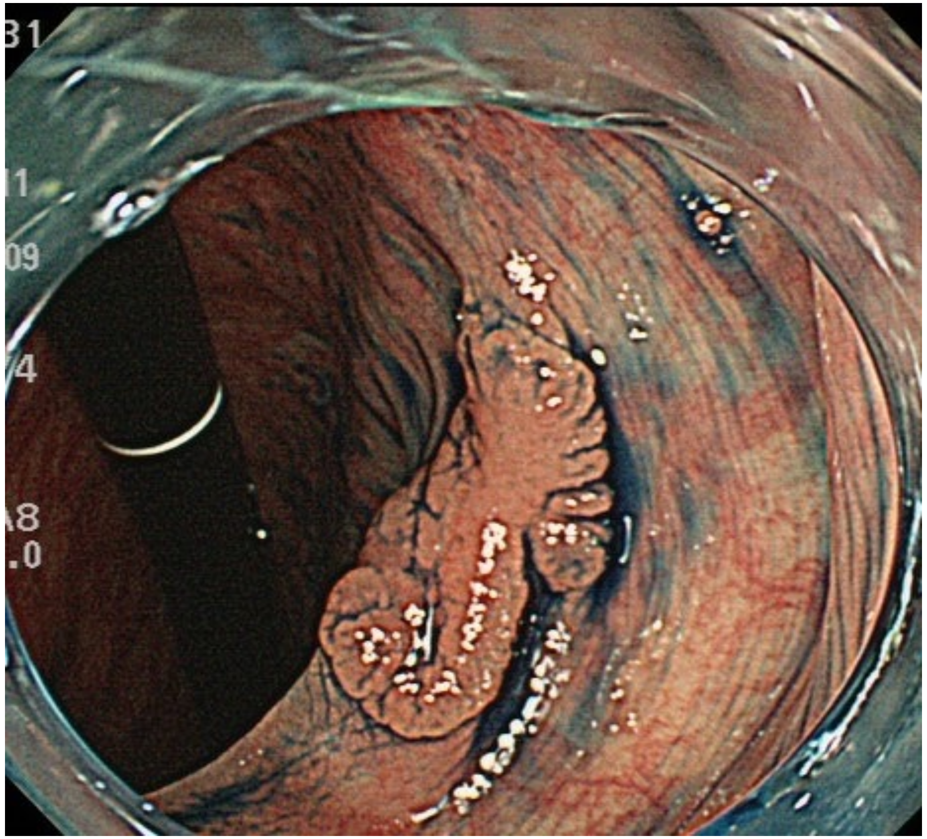

Q.　What is the morphological subclassification of this LST ?

Question ⑦

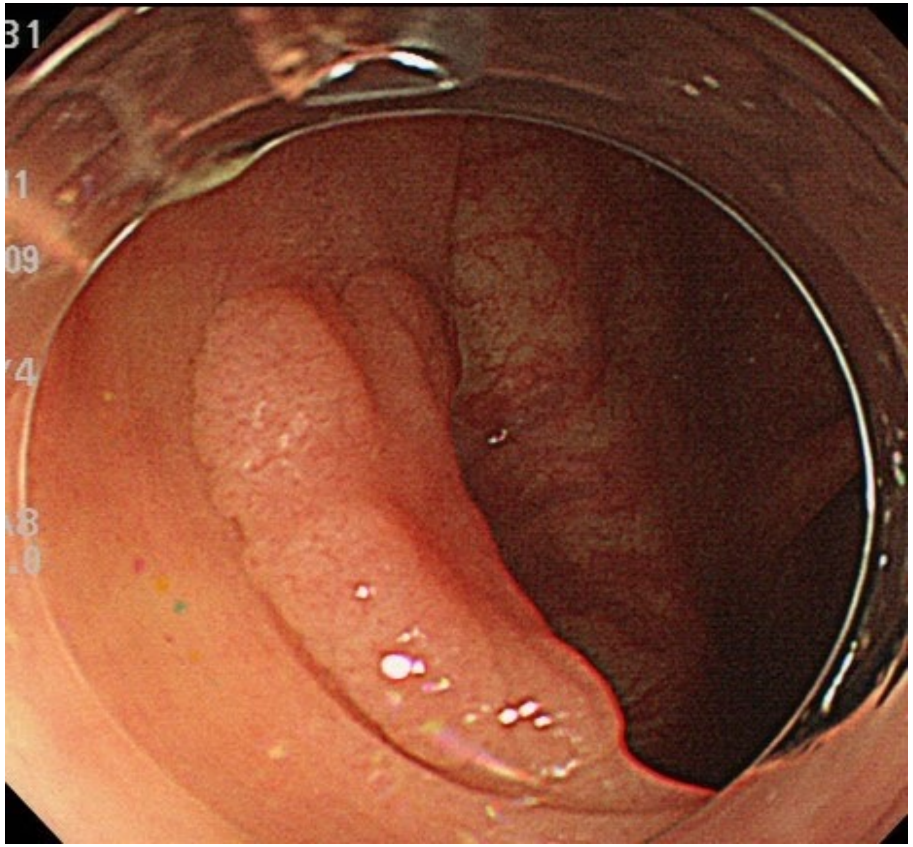

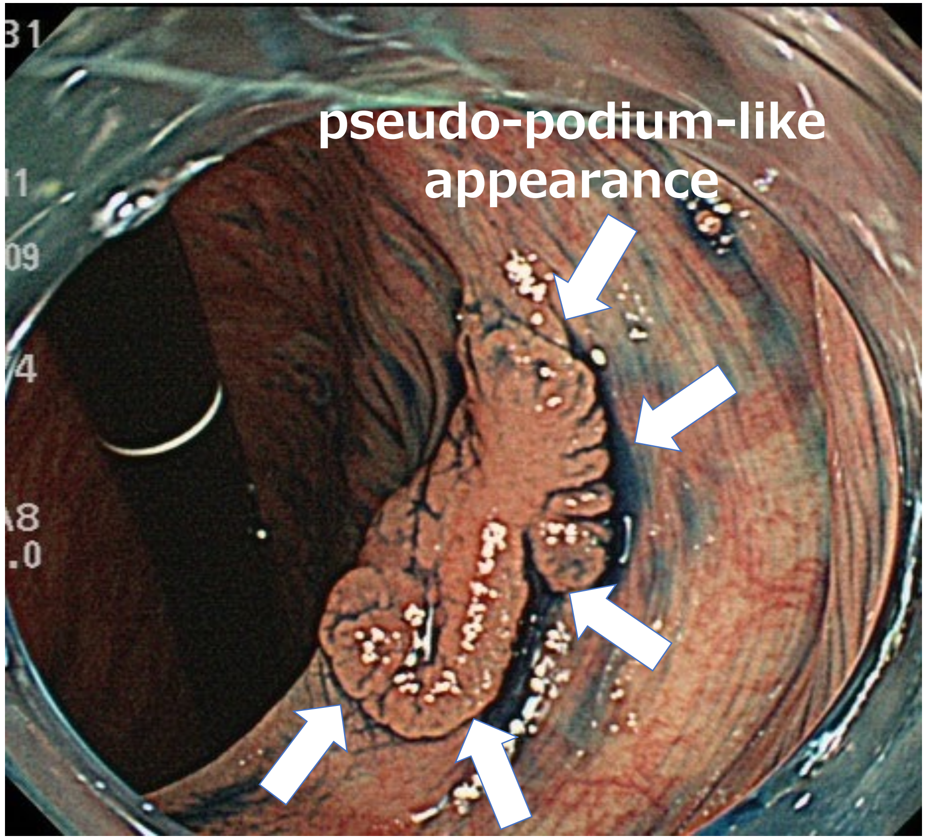

A. **LST-NG (PD)** (No granules. The lesion has pseudo-podium-like appearance.)
