# Supplementary material for: Use of a short educational video to improve the accuracy of colorectal polyp morphology assessment: A multicenter randomized controlled study
Source: DEN Open. 2025 Feb 3;5(1):e70066. doi: 10.1002/deo2.70066 (PMC11791016; doi:10.1002/deo2.70066)
Supplement: Supplementary file 2 — TABLE S1. Changes in pre‐test and diagnostic test accuracy in the complexed type and the subtype of laterally spreading tumors. TABLE S2. Multivariate analysis of factors that contributed to accuracy improvement (>10%) after the educational video lecture among participants, excluding those with pre‐test scores >80%. [file DEO2-5-e70066-s003.docx]

**SUPPORTING INFORMATION**

**TABLE S1. Changes in pre-test and diagnostic test accuracy in the complexed type and the subtype of laterally spreading tumors**

|  | Pre-test  accuracy | Diagnostic test accuracy | Changes |
| --- | --- | --- | --- |
| Complexed type | | | |
| IIa+Is  n = 2 | 39.2% | 58.2% | + 19.0 points |
| Is+IIa  n = 1 | 40.5% | 28.4% | - 12.1 points |
| Ip+IIc  n = 1 | 49.1% | 53.4% | + 4.3 points |
| IIa+IIc  n = 3 | 31.6% | 46.6% | + 15.0 points |
| Is+IIc  n = 3 | 46.0% | 60.1% | +14.1 points |
| LST | | | |
| LST-G-H  n = 3 | 79.9% | 83.9% | + 4.0 points |
| LST-G-NM  n = 3 | 85.6% | 92.0% | + 6.4 points |
| LST-NG-FE  n = 3 | 92.5% | 77.3% | - 15.2 points |
| LST-NG-PD  n = 3 | 53.2% | 79.3% | + 26.1 points |

IIa; Flat elevated

Is; Sessile

Ip; Pedunculated

IIc; Flat depressed

LST; Laterally spreading tumor

LST-G-H; laterally spreading tumor - granular - homogenous type,

LST-G-NM: laterally spreading tumor, granular, nodular mixed type

LST-NG-FE, laterally spreading tumor-non-granular-flat elevated type; LST-NG-PD, laterally spreading tumor-non-granular-pseudo-depressed type.

**TABLE S2. Multivariate analysis of factors that contributed to accuracy improvement (>10%) after the educational video lecture among participants, excluding those with pre-test scores >80%**

| Variable | | β | OR (95% CI) | *p*-value |
| --- | --- | --- | --- | --- |
| Classification of endoscopists | Non-beginners | 0.57 | 1.77 (0.60–5.22) | 0.30 |
|  | Beginners | Reference | Reference |  |
| Sex | Male | Reference | Reference | 0.87 |
|  | Female | -0.092 | 0.91 (0.31–2.66) |  |
| Number of colonoscopies conducted in the last year (n) | | 0.00 | 1.00 (1.00–1.00) | 0.067 |
| Pre-test score (%) | | –0.10 | 0.90 (0.86–0.94) | <0.001 |
| Number of views of the educational video (n) | | 0.47 | 1.60 (0.83–3.08) | 0.16 |

β: Regression coefficient, CI: Confidence interval, OR: Odds ratio
